# The Reactivity of CsPbBr_3_ Nanocrystals toward Acid/Base Ligands

**DOI:** 10.1021/acsnano.1c09603

**Published:** 2022-01-10

**Authors:** Francesco Zaccaria, Baowei Zhang, Luca Goldoni, Muhammad Imran, Juliette Zito, Bas van Beek, Simone Lauciello, Luca De Trizio, Liberato Manna, Ivan Infante

**Affiliations:** ^†^Department of Nanochemistry, ^§^Analytical Chemistry Lab, and ^∥^Electron Microscopy Facility, Istituto Italiano di Tecnologia, Via Morego 30, 16163 Genova, Italy; ‡ Dipartimento di Chimica e Chimica Industriale, Università degli Studi di Genova, Via Dodecaneso 31, 16146 Genova, Italy; ⊥ Department of Theoretical Chemistry, Faculty of Science, Vrije Universiteit Amsterdam, de Boelelaan 1083, 1081 HV Amsterdam, The Netherlands

**Keywords:** CsPbBr_3_, colloidal nanocrystals, density functional theory, ligand stripping, surface chemistry, stability

## Abstract

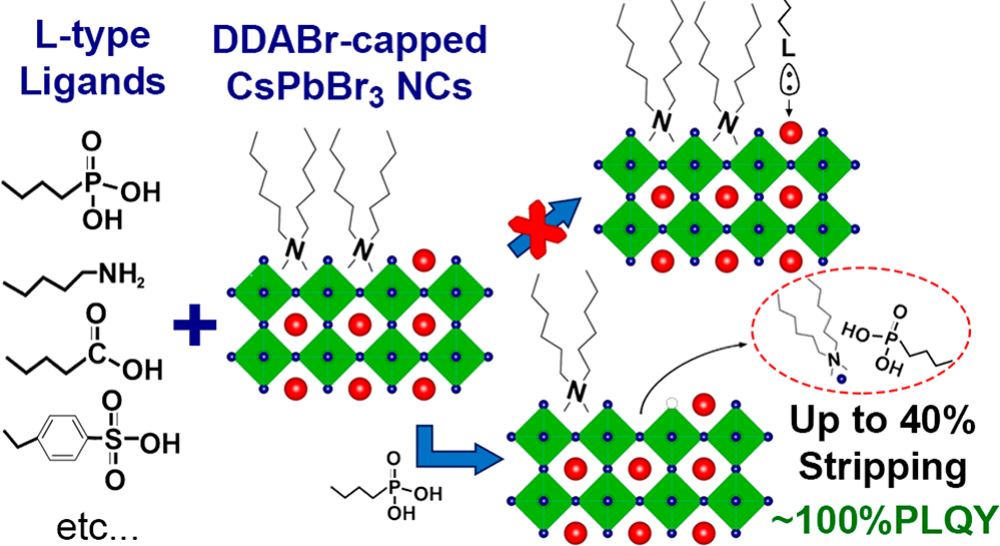

The interaction of lead bromide perovskite nanocrystals with charged ligands, such as salts, zwitterions, or acid–base pairs, has been extensively documented over the past few years. On the other hand, little is known about the reactivity of perovskite nanocrystals toward neutral ligands. To fill this gap, in this work we study the interaction of CsPbBr_3_ nanocrystals passivated with didodecyldimethylammonium bromide (DDABr) toward a series of exogenous acid/base ligands using a combined computational and experimental approach. Our analysis indicates that DDABr-capped nanocrystals are inert toward most ligands, except for carboxylic, phosphonic, and sulfonic acids. In agreement with the calculations, our experimental results indicate that the higher the acidity of the ligands employed in the treatment, the more etching is observed. In detail, dodecylbenzenesulfonic acid (p*K*_a_ = −1.8) is found to etch the nanocrystals, causing their complete degradation. On the other hand, oleic and oleylphosphonic acids (p*K*_a_ 9.9 and 2, respectively) interact with surface-bound DDA molecules, causing their displacement as DDABr in various amounts, which can be as high as 40% (achieved with oleylphosphonic acid). Despite the stripping of DDA ligands, the optical properties of the nanocrystals, as well as structure and morphology, remain substantially unaffected, empirically demonstrating the defect tolerance characterizing such materials. Our study provides not only a clear overview on the interaction between perovskite nanocrystals and neutral ligands but also presents an effective ligand stripping strategy.

## Introduction

Lead halide perovskite nanocrystals (NCs) with the general formula APbX_3_ (A = Cs^+^, CH_3_NH_3_^+^, CH(NH_2_)_2_^+^ and X = Cl^–^, Br^–^, I^–^) is a family of band gap tunable semiconductors highly sought for optoelectronic and photonic applications.^1−14^ Although this class of materials is often considered to be defect tolerant, a proper surface passivation remains the key to achieve highly efficient and stable emitters.^15−17^ Therefore, understanding their surface chemistry, with a pivotal focus on the interplay between NC surface and the ligands shell, is fundamental for their implementation in technological applications.^17−20^

In the family of lead halide perovskites, CsPbBr_3_ NCs can be prepared with desired size, shape, and surface composition (for instance with either CsBr- or PbBr_2_-terminated surfaces).^6,21^ Their colloidal synthesis typically relies on the use of primary amines and carboxylic acids (*i*.*e*., oleylamine and oleic acid) as surfactants, which bind to the surface of the NCs in the form of alkylammonium-Br and Cs-carboxylates, respectively.^22−24^ Perovskite NCs bearing such surface passivation are characterized by a poor stability, since their purification, storage, or exposure to air easily leads to protonation/deprotonation of carboxylate/alkylammonium species, with their consequent desorption from the surface.^25,26^ With the aim of mitigating these problems, several postsynthetic ligand exchange strategies have been developed. These reactions usually consist in the replacement of Z-type ligands (alkylammonium-Br and Cs-carboxylates) with new Z-type ones, such as quaternary ammonium halides or inorganic salts containing pseudo halide anions (SCN^–^, BF^4–^).^27−29^ These procedures can lead to a marked improvement of the NCs stability, but they can also trigger drastic structural and compositional transformation of the host NCs if the amount of added species is not well calibrated.^30−32^

While substantial progress has been made in ligand exchange reactions employing Z-type ligands, a systematic study on the interactions between the surface of halide perovskite NCs and neutral ligands (L-type) is still lacking. For example, it is not known if Z-type ligands can be replaced by L-type ligands, as in the case of “classical” II–VI colloidal systems,^33,34^ or if L-type ligands can bind to free sites (*i*.*e*., those not occupied by Z-type ligands) on the NC surface to further enhance their stability, as also indicated in some recent works.^35,36^ This is mainly due to the fact that such interactions have to be probed in completely aprotic conditions, which are not easily achieved. In fact, even when the working environment is air- and moisture-free and only aprotic solvents are employed, perovskite NCs are typically covered by ligands that can be involved in rapid proton exchange reactions with added neutral ligands. For example, a protonated (or deprotonated) native ligand such as RNH_3_^+^ (or RCOO^–^) may donate (or accept) a proton to (from) a neutral free ligand, becoming itself a neutral species and be displaced from the surface of the NCs.^22,24,31^

To circumvent such problems, we have developed here an *ad-hoc* system to probe the interaction of CsPbBr_3_ NCs with neutral ligands in a completely aprotic environment. In detail, we employed CsPbBr_3_ NCs coated by didodecyldimethylammonium bromide (DDABr) species only, that is, ligands that cannot lose or acquire protons.^25^ Such NCs were then exposed to a series of organic ligands (listed in Table 1) with neutral head groups of varying acidity/basicity under strictly anhydrous conditions. We denote these ligands as *exogenous* to distinguish them from those already bound to the starting NCs. More specifically, based on a combination of density functional theory (DFT) calculations and experiments, we tested whether these exogenous ligands can interact with the NCs, promoting either a simple detachment of the native ligands or more drastic effects such as etching, dissolution, or phase transformations. With this combined approach, we demonstrate that, under aprotic conditions, most neutral species tested did not interact with the DDABr-capped NCs even when employed in large excess, except for oleic acid (HOA), oleylphosphonic acid (OLPA), and dodecylbenzenesulfonic acid (DBSA). We purposely chose these latter ligands because they present a significant change of acidity, from moderate to high values. In this way, a change in the chemistry of the perovskite system can be traced more easily from both a theoretical and computational standpoint. The resulting etching degree of these molecules was indeed found to follow their acidity, as also shown by calculations. Indeed, while DBSA, the strongest acid employed here, led to the complete etching of the NCs, HOA and OLPA were observed to interact as L-type ligands with a fraction of the bound DDA molecules, stripping them from the NC surface (most likely in the form of DDABr). In particular, the treatment with OLPA was more effective than HOA (as the former is more acidic than the latter) in stripping ligands, leading to the removal of 40% of starting DDA ligands, while preserving the optical properties and colloidal stability of the NCs. To the best of our knowledge, this result represents not only an empirical evidence of the defect tolerance of CsPbBr_3_ NCs but also a documented case of a controlled ligand stripping approach to halide perovskite NCs. This outcome, in turn, leads to the fabrication of devices based on colloidal perovskite NCs, where the amount of insulating ligand species must be minimized, while retaining the optical efficiency and colloidal stability to work with stable inks (especially if the devices are produced by ink jet technologies).

**Table 1:** Neutral Ligands Considered and Relative Abbreviation of Their Names

protic ligands (p*K*_a_)	abbreviation
HBr (−9)	HBr
dodecylbenzenesulfonic acid (−1.8)	DBSa
oleylphosphonic acid (2)	OLPA
dioctylphosphinic acid (2)	DOPa
oleic acid (9.9)	HOA
octylthiol (10)	OcS
octanol (16)	OcOH
octylamine (40)	OcN
dioctylamine (40)	DON

aprotic ligands	abbreviation
dioctylsulfide	DOS
trioctylamine	TON
trioctylphosphine	TOP
trioctylphosphine oxide	TOPO
octanal	OcH
dimethylsulfoxide	DMSO

## Results/Discussion

### Preparation of the CsPbBr_3_ Model System

**Figure 1:**
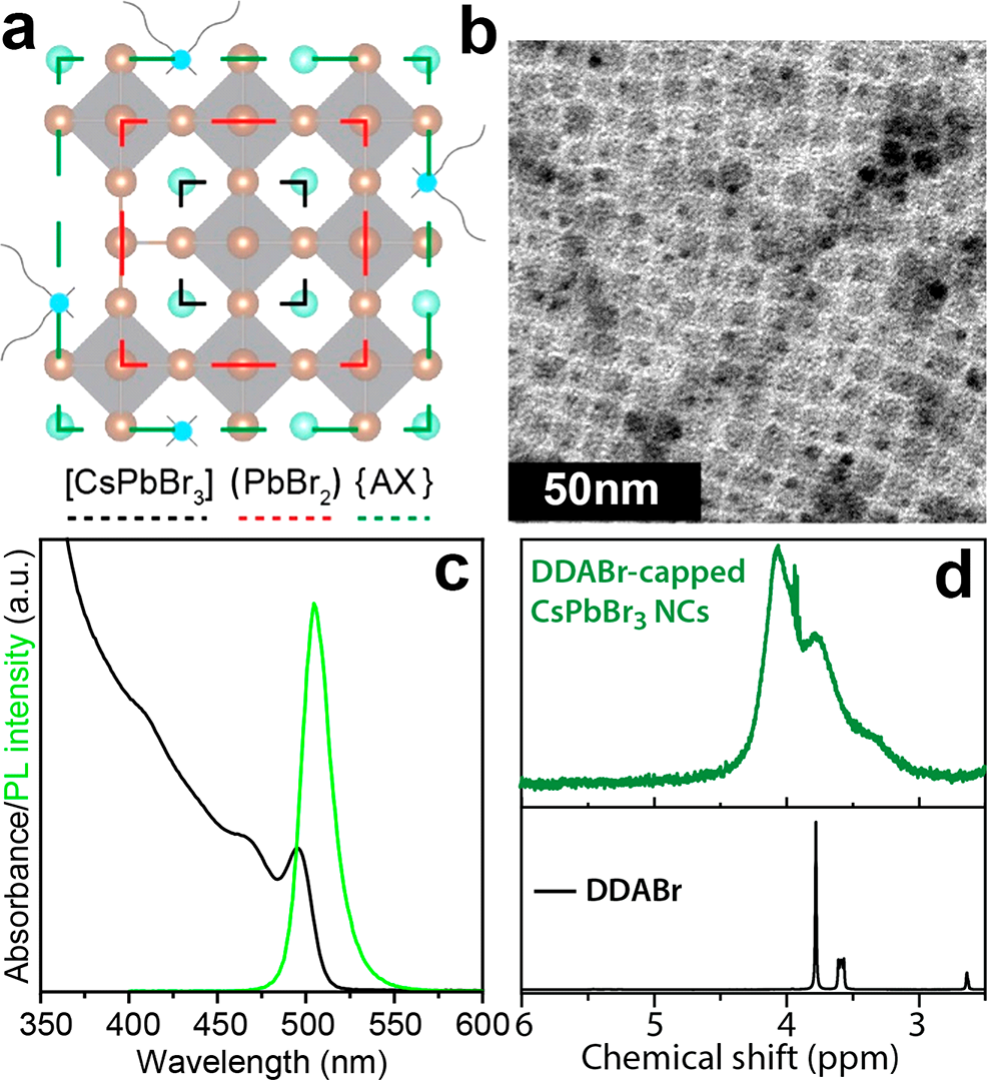
(a) Sketch depicting the structure of a DDABr-capped CsPbBr_3_ NC. (b) TEM picture, (c) absorption and PL spectra (λ_em_ = 504 nm), and (d) ^1^H NMR spectrum (collected in toluene-*d*_8_) of DDABr-capped CsPbBr_3_ NCs.

Colloidal CsPbBr_3_ NCs capped with DDABr (Figure 1a) were prepared by following a recently published procedure by our group (see the Methods section for additional details).^25^ Such NCs are prepared under an inert atmosphere and without protic solvents to guarantee a completely proton-free environment. These NCs will set the reference for all our surface treatments with neutral ligands. As shown in Figure 1b, the NCs have a cubic shape and are nearly monodisperse in size, with an average edge length of 5.9 ± 0.7 nm (Figure S1 of the Supporting Information). Their absorption and photoluminescence (PL) spectra are reported in Figure 1c, and their PL quantum yield (QY) is 84%. As shown in Figure 1d, the ^1^H NMR spectrum of such NCs in toluene-*d*_8_ features two broad signals in the 4.1–3.5 ppm range belonging to surface-bound DDA molecules.^37^ The chemical shift of these two peaks, at lower fields compared to those of free DDABr molecules (characterized by two sharp peaks at 3.9 and 3.7 ppm, see Figure 1d), indicates that the electron density of the bound species around the ^1^H nuclei of DDA is lowered by the interaction with the surface of the NCs.^38^ Signal broadening is due to ligands interacting with the NCs’ surface, which causes a slower mobility in solution, *i*.*e*., with longest correlation times (τ_c_), compared to correspondent free ligands.^38^

The composition of the inorganic core of the NCs was measured *via* energy dispersive X-ray spectroscopy (EDS) in the scanning electron microscope (SEM). On the other hand, the quantification of the bound ligand species was assessed by NMR analysis after dissolving the NCs in deuterated dimethyl sulfoxide (DMSO, see the Methods section for details).^39^ The combination of these analyses indicated that the NCs are terminated by a PbBr_2_ inner shell and are capped by a hybrid AX outer shell (A = DDA, Cs; X = Br, oleate), as depicted in Figure 1a (see Method S1 and Table S1 for further details). Assuming that the core and inner shell regions are pristine, by following the [core](inner-shell){outer-shell} nomenclature proposed by Bodnarchuk et al.^26^ the composition of the NCs can be conveniently written as
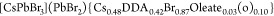


In the formula above, the AX outer layer is composed of two types of A cations (Cs^+^ and DDA^+^) and two types of X anions (Br^–^ and Oleate^–^), with the addition of AX surface vacancies defined as (o). This overall composition corresponds to a surface (outer shell) coverage (occupation) of up to 90%, in line with the high photoluminescence quantum yield (PLQY) observed from these NCs. Although there is a residual fraction of oleate ligands bound to the surface of the NCs, this fraction is essentially negligible, and henceforth we will refer to these NCs as DDABr-capped NCs.

### Surface Reactivity of the CsPbBr_3_ Nanocrystals from a Computational Perspective

Possible reactions between NCs and the exogenous organic ligands listed in Table 1 include simple adsorption, as well as chemisorption and etching (that may lead to phase transformations). Here, by chemisorption we mean the process by which an initially neutral exogenous ligand becomes charged (through the loss of one of its protons) and binds to the surface of the NCs as a charged species. For simplicity, in our calculations we consider all the A cations to be Cs only, unless otherwise stated. Although the complete absence of DDA ligands on the surface of NCs is not realistic, our computations describe the outer shell in its fully inorganic form as the only way to promote computational consistency and avoid effects, such as ligand–ligand interactions, that are difficult to estimate in the calculations. Additionally, for simplicity, in some cases we have considered ligands with a shorter ligand chain with respect to those listed in Table 1 (*i*.*e*., for the description of oleic acid, oleylphosphonic acid, and dodecylbenzyl sulfonic acid we used octanoic acid, Oca, octylphosphonic acid, OcPa, and, ethyl benzene sulfonic acid, EBSa).

**Figure 2:**
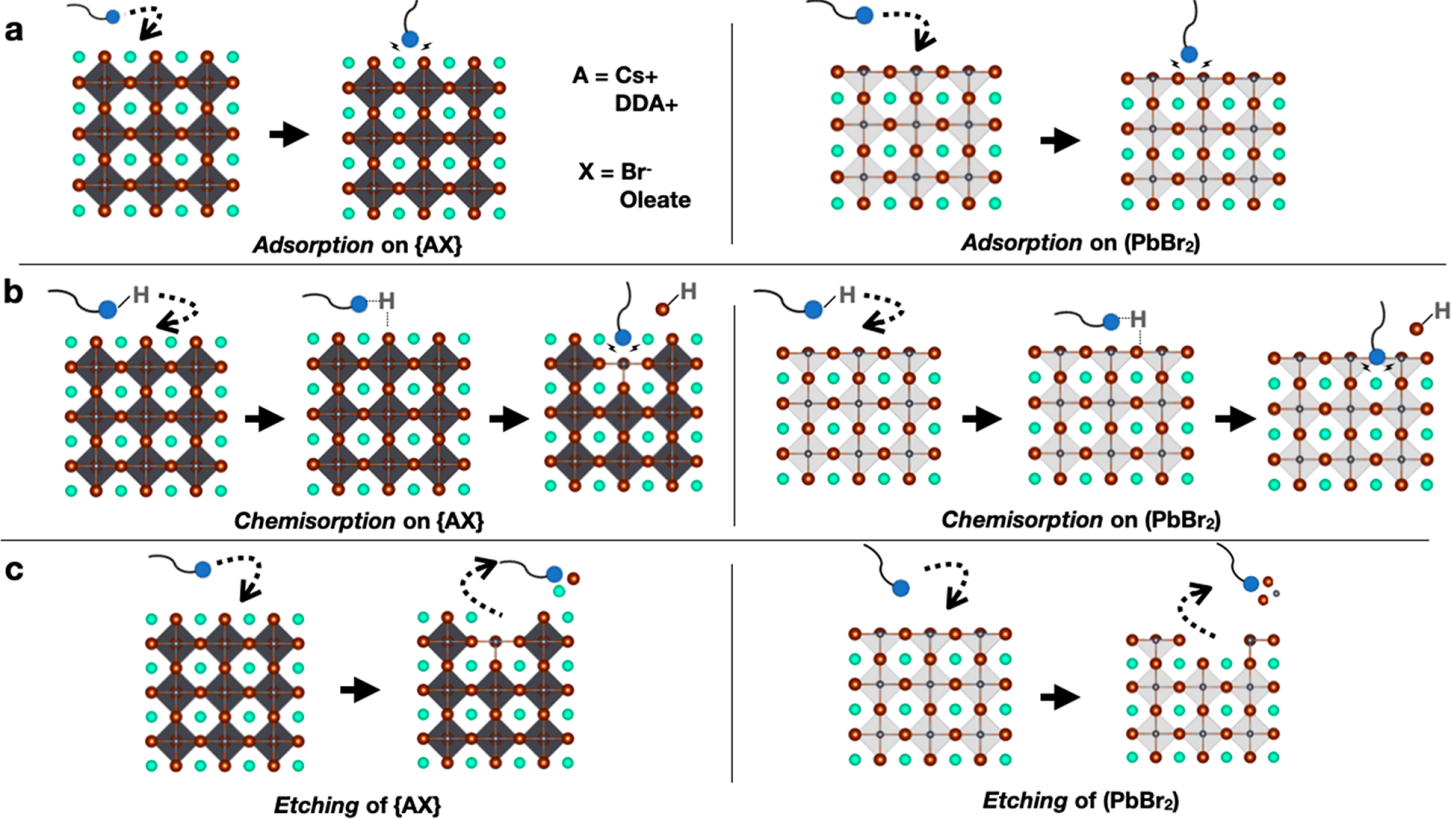
(a) Representation of direct adsorption of a neutral ligand on the outer {AX} or inner PbBr_2_ shell. (b) Proton-induced ligand exchange, *i*.*e*., chemisorption, describes the event of protonation of the {AX} surface by a protic ligand: the incoming ligand passivates the {AX} surface and causes the removal of an anion that leaves the surface in its protonated form. On the right, the same description for the (PbBr_2_) shell. (c) The interaction of a neutral ligand with the outer or inner shell culminates with the etching of an ion pair or a Z-type ligand.

All the considered surface reactions are schematically represented in Figure 2, whereas the correspondent chemical equilibria are described in more detail in the Supporting Information (Method S2). We note that favorable energetics for the removal of both ABr and PbBr_2_ units may be indicative of NC dissolution, while the preferential removal of one unit *in lieu* of another could explain phase transformations, common in the Cs–Pb–Br system, and well known in the field of halide perovskites.^40^ The energetics of the surface reactions is computed for all ligands listed in Table 1 using a 3 nm CsPbBr_3_ NC and employing the equations defined in the methodology section.

### Adsorption

#### Direct Ligand Absorption on {AX} and (PbBr_2_) Shells

**Figure 3:**
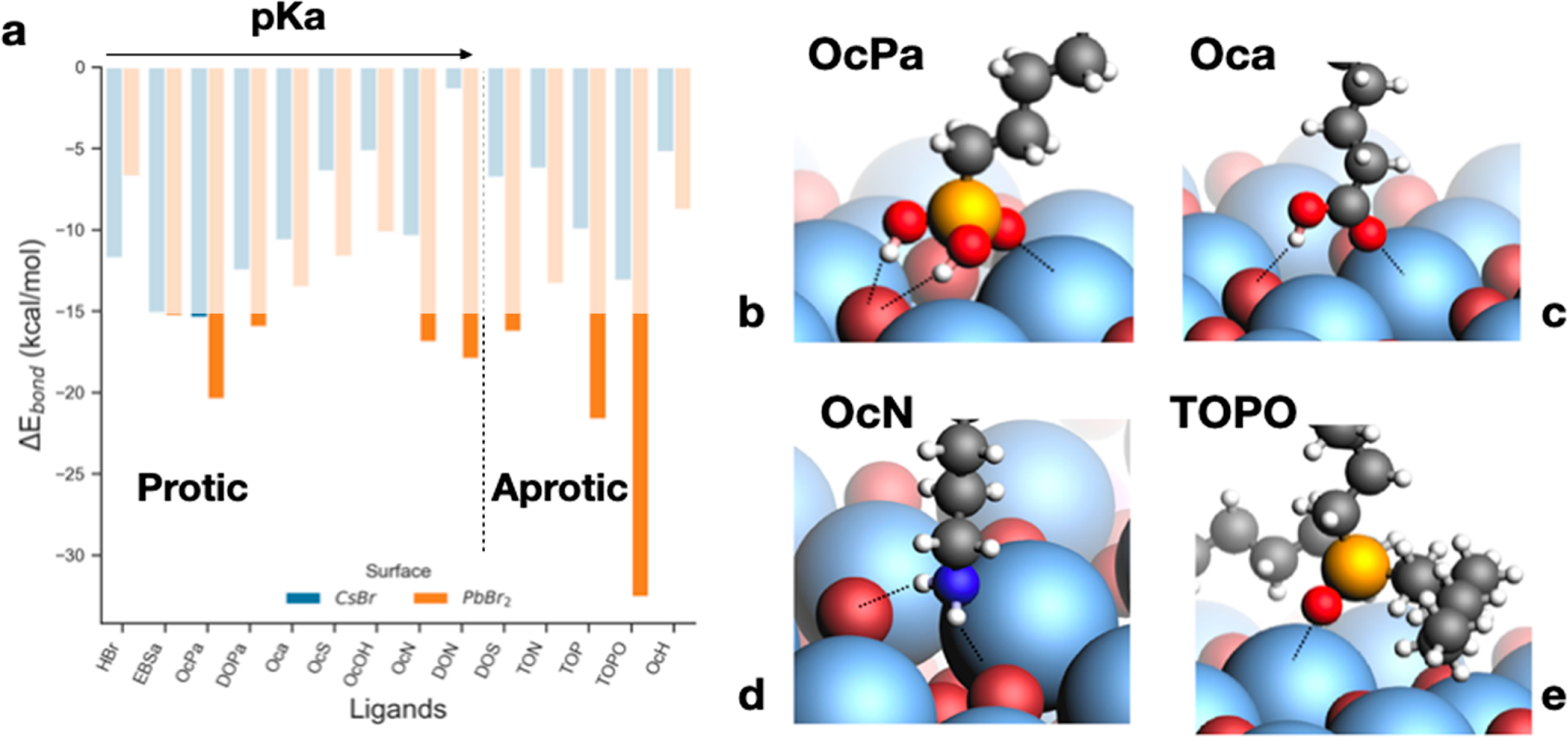
(a) Adsorption energy of neutral ligands on a bare CsBr (blue) or PbBr_2_ (orange) NC surface. Values, expressed in kcal/mol, refer to the interaction of an isolated ligand on the outer or inner shell of the NC. Values outside the shaded regions indicate the possibility of overcoming the entropic energy penalty. This means that only ligands in the nonshaded area effectively bind to the surface. (b) Sketches of the binding features for octyl phosphonic acid (OcPa), octanoic acid (Oca), octylamine (OcN), and trioctylphophine oxide (TOPO). OcPa and Oca are representative of oleyl phosphonic acid (OLPA) and oleic acid (HOA).

By adding exogenous neutral ligands to a colloidal suspension of NCs coated with native ligands, we can expect that their headgroups interact with the NC surface by attacking available surface binding sites, either on the available A^+^ or Br^–^ ions on the outer shell or on Pb^2+^ and Br^–^ on the inner shell (*i*.*e*., onto vacant sites). The binding of neutral protic ligands usually involves hydrogen bonding between the proton(s) located on the protic ligand’s anchoring group and the Br anions, with the ligand’s headgroup pointing toward the positive A or Pb sites of the outer and inner shells, respectively. The binding of aprotic (basic) ligands occurs through the interaction of the ligand’s headgroup directly on the A or Pb site. Calculated adsorption enthalpies are plotted in Figure 3a. Sketches of these binding features are displayed in Figure 3b–e. These results show that adsorption is exothermic for all ligands considered and that adsorption onto the PbBr_2_ surface (∼10–35 kcal/mol) is generally more favorable than onto the CsBr surface (∼2–15 kcal/mol). The binding energies of protic ligands roughly follows the same trend of their p*K*_a_, with more acidic ligands being more strongly bound to the surface. An exception is octylamine (OcN), which, despite high p*K*_a_ values, interacts well with the CsBr surface due to two hydrogen bonds and with the N atom pointing favorably toward a Cs^+^ ion at the surface (Figure 3d).

The adsorption energies computed above account only for binding enthalpies. The addition of an entropic contribution involves the loss of translational and rotational entropy. This in turn would entail, in principle, a significant energy penalty, only partly counterbalanced by an increase in the vibrational entropy (see for more details the Computational Methodology section). Ultimately, we estimate that the total energy penalty due to entropic contribution is in the interval 10–15 kcal/mol. On the basis of these qualitative observations and looking again at Figure 3a, we can expect that for ligands binding to the {AX} outer shell, the entropic penalty renders the free energies of adsorption slightly endergonic, whereas for the (PbBr_2_) inner shell we can expect that some ligands could still bind to the NC surface. These observations are in line with a recent report showing that the addition of stoichiometric amounts of phosphonic acid (namely, oleylphosphonic acid) to a dispersion of CsPbBr_3_ NCs having PbX_2_-terminated facets results in the surface binding of the acid in its neutral state.^35^

### Chemisorption

#### Proton-Induced Ligand Exchange

**Figure 4:**
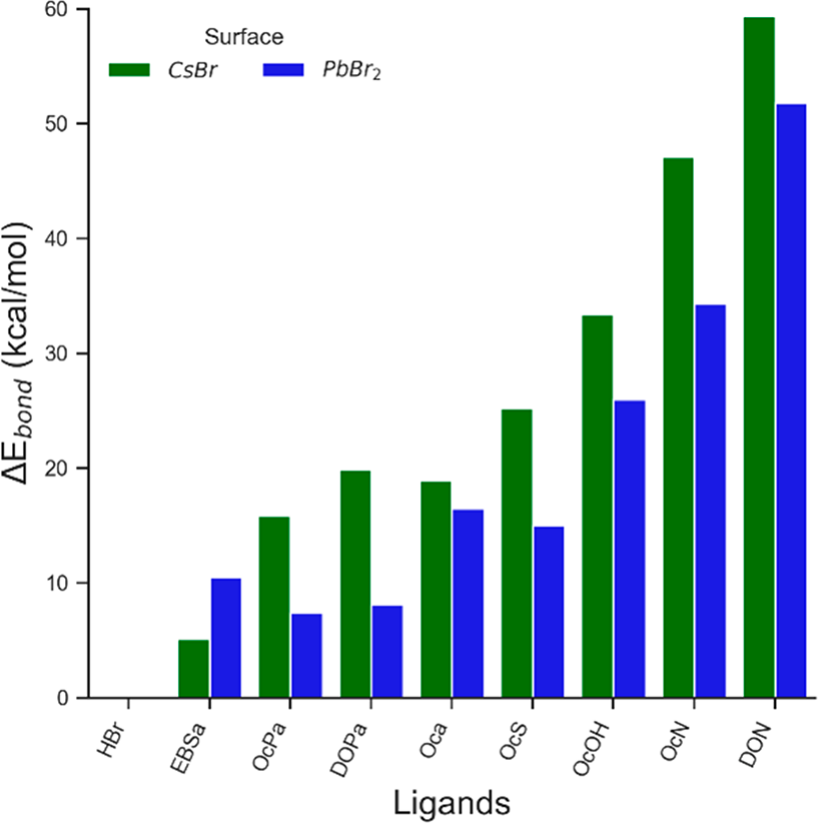
Energies related to events of proton-induced ligand exchange on both {AX} and (PbBr_2_) surfaces. The graph shows how protic ligands are unable to displace bromide ions as HBr because this leaving group is the strongest acid of the series.

The second process we investigated is the proton-induced ligand exchange. Since we have chosen the DDA^+^ ligand to passivate cation sites, the only process eventually involving a proton transfer can occur when a *protic* ligand (HL) donates its proton to a surface anion (Br^–^), promoting the desorption of HBr and the adsorption of L^–^. The computed enthalpies of chemisorption for protic ligands onto CsPbBr_3_ NCs are shown in Figure 4. The trend follows roughly that of p*K*_a_, with stronger acids (lower p*K*_a_ values) yielding lower enthalpy values. In any case, positive enthalpy changes for all the organic ligands indicate their inability to displace Br^–^ ions in the form of HBr, since the p*K*_a_ of such ligands is higher than that of HBr. Our results also show that the enthalpic cost of chemisorption is generally slightly lower on PbBr_2_ surfaces. Here we assume that the enthalpic contribution is dominant, with only a small contribution from the entropy, as the number of species is the same at both sides of the chemisorption reaction.

Although not strictly related to our reference NC model, we also considered the protonation process involving functionalized surfaces, *e*.*g*., protonation of the residual carboxylate moieties (*e*.*g*., oleate) bound to the surface of the NCs. This determines the release of the corresponding carboxylic acid, allowing for the insertion of the exogenous ligand, as anion, to the surface. In our calculations, octanoate (emulating the oleate), as the conjugated base of an acid with a p*K*_a_ of ∼5, can be displaced by acids stronger than octanoic such as phosphonic, sulfonic, and halic acids (see Figure S2). Very weak acids, such as amines, will not trigger any ligand exchange. It is interesting to note that the trend for the ligand exchange follows closely the trend in p*K*_a_, although the medium in which these chemical processes take place is an organic solvent with a low dielectric constant. The key point is that, in an apolar solvent, the NC itself accepts protons and stabilizes the conjugated bases resulting from the dissociation of protic ligands, thus effectively mimicking the behavior of an amphoteric solvent.

### Etching

#### Ligand-Induced Displacement of {ABr} and (PbBr_2_) Shells

**Figure 5:**
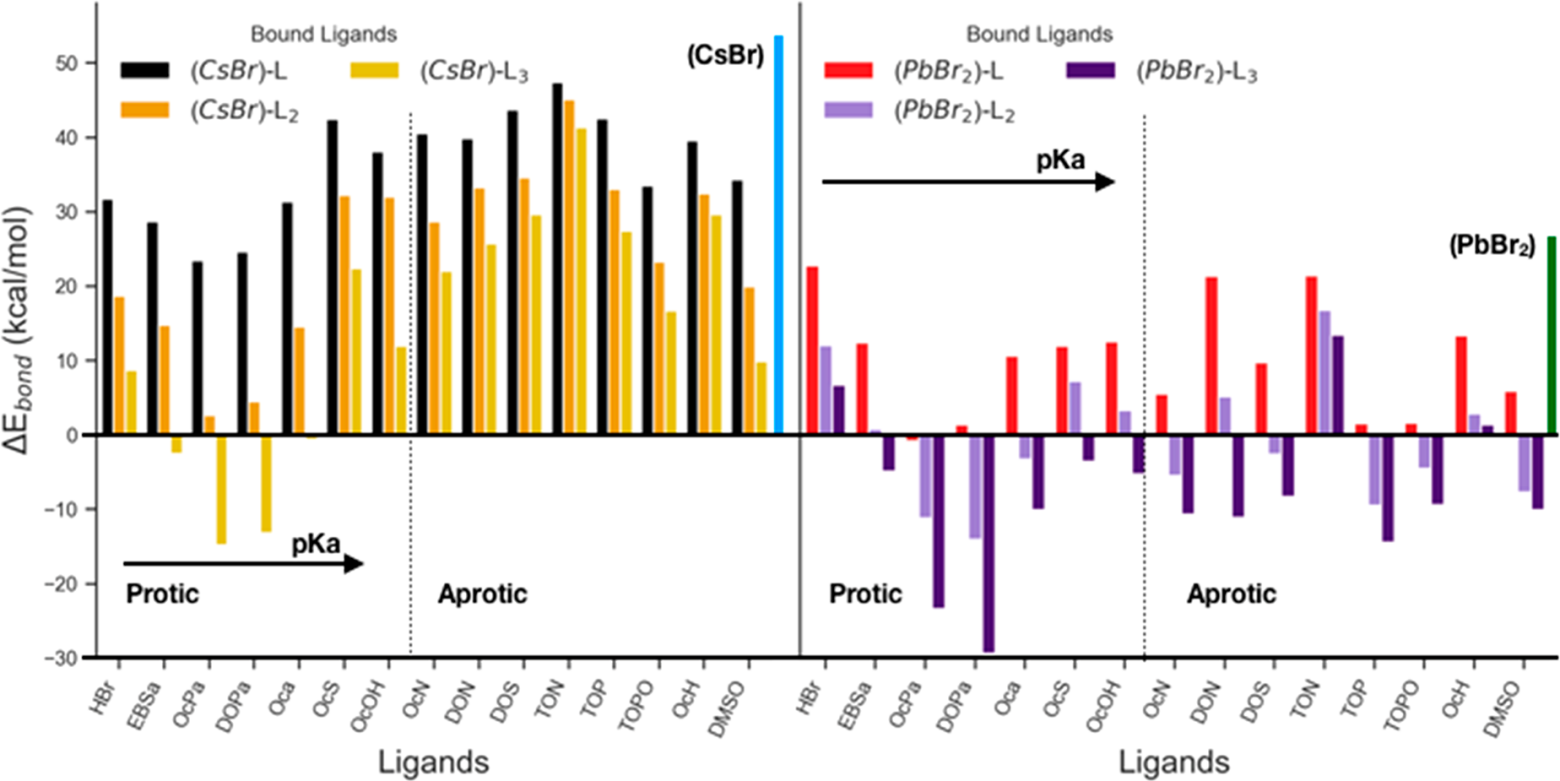
Ligand-induced displacement of CsBr ion pairs and PbBr_2_, after interaction of neutral ligands with bare surfaces. The presence of any of the investigated ligands lowers the energy required to displace CsBr and PbBr_2_ shells. Excess ligand can further stabilize the leaving moieties: this is particularly relevant when excess ligand interacts with (PbBr_2_) shells, where almost in all cases, theoretically, the presence of 3 ligands per PbBr_2_ would strongly favor the displacement of the latter.

The last process we discuss is the ligand-induced displacement of ion pairs from the NC surface: this resembles the Z-type ligand-induced displacement processes occurring in II–VI, III–V, and IV–VI NCs.^33,34,41^ First, we note that the simple desorption, *i*.*e*., not assisted by ligands, of a CsBr ion pair from the NC surface is highly endothermic, requiring *ca*. 52 kcal/mol (blue bar in Figure 5) based on our calculations. In other words, a surface CsBr unit is strongly bound to the NC (even when considering 10–15 kcal/mol of entropic penalties that favors the displacement). Other AX ion pairs, representative of most used CsPbBr_3_ passivating ligand pairs, such as DDABr, cesium octanoate, ammonium bromide, and ammonium octanoate, are also found to be strongly bound to the surface, although slightly less than CsBr, with energies of the same order of magnitude (>44 kcal/mol, see Figure S3).

In the case of ligand-induced displacement reactions (see Method S2), we expect entropic penalties to be small, and Δ*G* ∼ Δ*H*. The enthalpies of these reactions are plotted in Figure 5. It is worth noting that the enthalpy of displacement decreases with increasing number of ligands per displaced moiety (CsBr or PbBr_2_ units). In fact, at 1 ligand per displaced moiety the calculated enthalpy changes are endothermic for most ligands, but they become exothermic as the number of ligands is increased (up to 3 ligands per moiety). This effect is particularly strong for strong acids, with reductions of up to 30 kcal/mol being observed, while for basic ligands the reductions are in the 5–15 kcal/mol region. Furthermore, we observe that displacing a PbBr_2_ unit is energetically more favorable than displacing a CsBr unit. The non-ligand-assisted displacement of a PbBr_2_ unit costs ∼28 kcal/mol, which is about half of the energy required to remove CsBr (∼52 kcal/mol). Displacing a PbBr_2_ unit with ligands is similarly more favorable. Among all ligands, phosphinic and phosphonic acids exhibit the most favorable energetics for displacing both PbBr_2_ and CsBr units, suggesting that these ligands may undermine the integrity of the NCs. Basic ligands, on the other hand, appear unable to detach CsBr units, although some of them can extract PbBr_2_ units (trioctylphosphine and trioctylphosphine oxide in particular). These findings apparently do not seem to be in line with the phase transformations observed in Cs–Pb–Br NC systems, namely, the CsPbBr_3_ → Cs_4_PbBr_6_ transformation, which is triggered by treating the NC with primary amines.^32,42^ This process is instead induced by the protonation of the added primary amine operated by moisture/native ligands, as it will be discussed later in this work (see also Figure S4).

Finally, we also compared the ligand-induced displacement of other common {AX} pairs such as DDABr, DDA-octanoate, and Cs-octanoate (Figure S3). Also in this case, the energetics of displacement resembles the trends found in the CsBr case, further confirming that the enthalpies of etching are somehow independent of the native ligands capping the {AX} outer shell (*i*.*e*., independently of their inorganic or fully organic nature).

### Experimental Results

**Scheme 1:**
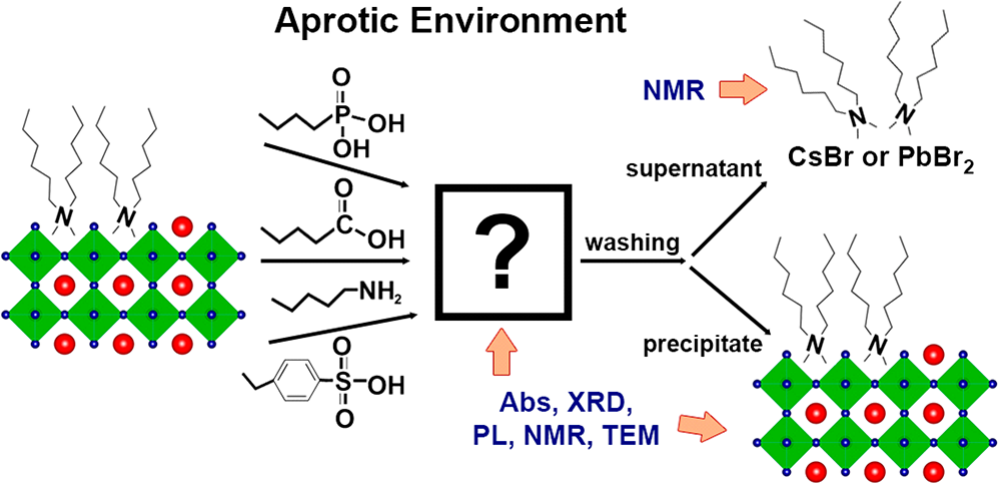
Experimental procedure and characterization of the products.

We tested the reactivity of DDABr-capped NCs toward most of the neutral ligands listed in Table 1 except for HBr, DMSO, and octanol, as these are known to dissolve perovskite NCs.^6^ Moreover, we selected OLPA and HOA as representative of carboxylic and phosphonic acids, as they are easier to handle with respect to octanoic acid and octylphosphonic acid (the latter is solid and poorly soluble in toluene). To do so, NC dispersions in toluene ([NCs] ≈ 16 μM) were treated with different amounts of exogenous ligands, ranging from 1 to 10 equiv with respect to surface Br sites (considering 726 surface Br per NC, the concentration of surface sites in solution was calculated to be 7.8 mM; see Method S3 and Table S1). To avoid any moisture contamination, which could lead to deprotonation/protonation of the neutral ligands employed, all the operations were carried out in a N_2_-filled glovebox by using anhydrous solvents and ligands. The resulting products were thoroughly characterized optically, structurally, and chemically (see Scheme 1 and the Methods section).

**Figure 6:**
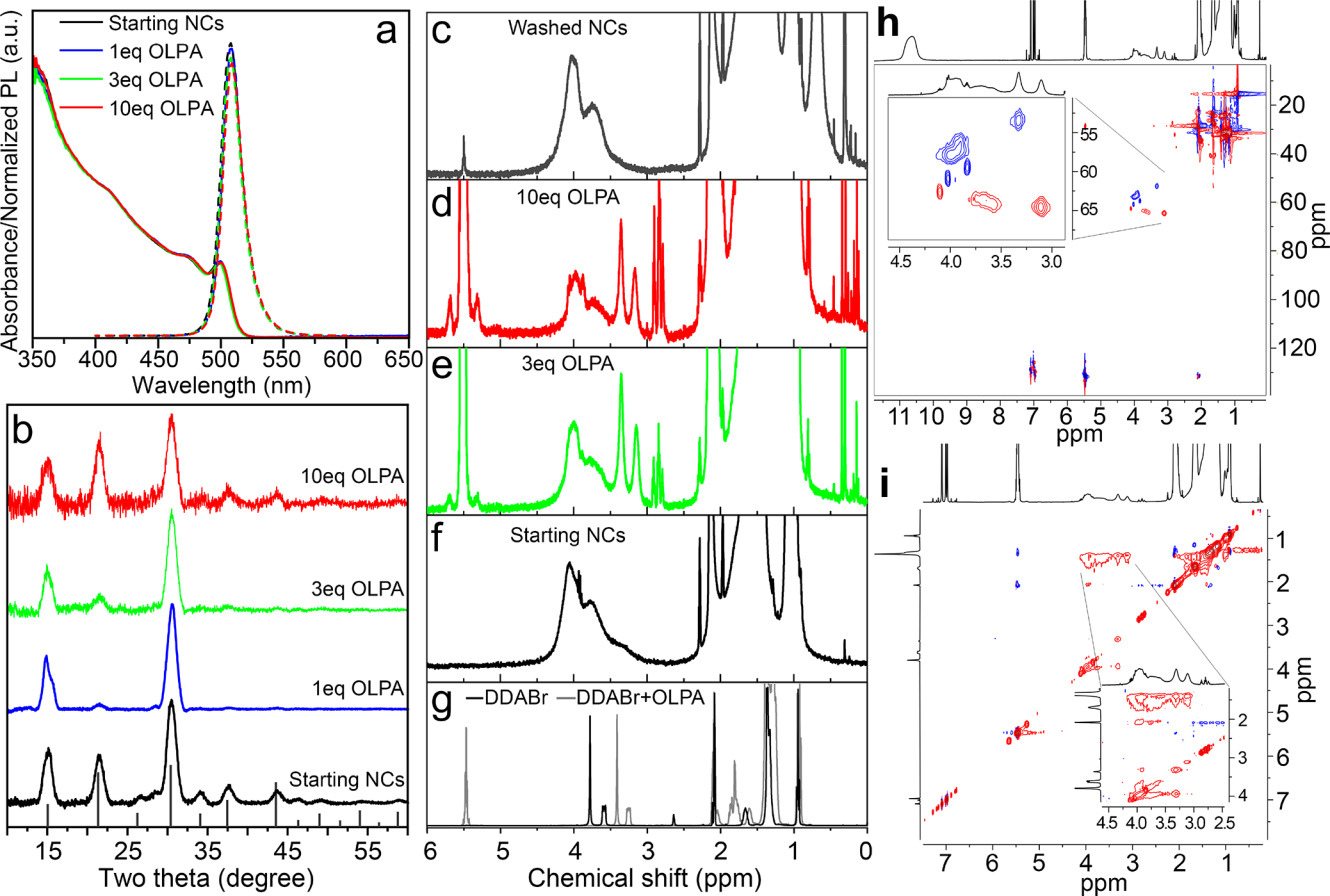
(a) Optical properties and (b) XRD patterns of DDABr-capped CsPbBr_3_ NCs treated with 1, 3, or 10 equiv of OLPA. ^1^H NMR spectra of (f) DDABr-capped CsPbBr_3_, (d, e) OLPA treated, and (c) washed (after treatment with 3 equiv of OLPA) NCs. (g) ^1^H NMR spectra of free DDABr molecules and DDABr with the addition of OLPA molecules. (h) Heteronuclear single quantum coherence spectroscopy (^1^H−^13^C HSQC) edited with the selection of CH_2_ (red) and CH/CH_3_ (blue) of DDABr-capped NCs treated with 3 equiv of OLPA in toluene-*d*_8_. The extra peak at 3.31 ppm (with the ^13^C at 53 ppm) was identified as CH_3_ (blue), whereas the extra peak at 3.11 ppm (with the ^13^C at 64.6 ppm) was associated with a CH_2_ (red). The ^13^C resonances are typical of CH_3_ and CH_2_ on nitrogen, respectively. (i) 2D ^1^H–^1^H NOESY experiment performed at 40 °C of DDABr-capped NCs treated with 3 equiv of OLPA in toluene-*d*_8_. OLPA returns positive NOE (blue) cross peaks, characteristic of species with a short correlation time (τ_c_), whereas DDABr broad signals at 3.96 and 3.74 ppm and the new extra peaks at 3.31 end 3.11 ppm exhibit negative (red) cross peaks, typical of species with long correlation times (τ_c_), indicating that those species are dynamically interacting with the surface of the NCs.

Our results show that DDABr-capped CsPbBr_3_ NCs are inert toward most of the ligands tested in this work, as shown in Figures S5 and S6. On the other hand, OLPA, HOA, and DBSA were observed to interact with the NCs. The exposure of the NCs to various concentrations of OLPA (1, 3, or 10 equiv) did not yield any significant difference in their optical properties (Figure 6a). Furthermore, XRD and TEM analyses of OLPA-treated NCs show that their structural and morphological features remained preserved (Figures 6b and S7). It should be noted here that, in some cases, the XRD peak at 15° appears as a doublet (see Figure 6b, blue pattern) as a consequence of a superlattice effect (*i*.*e*., the NCs organize in an ordered superlattice in the drop-casted films).^43,44^ To reveal if OLPA had any interaction with the surface of the NCs, we performed liquid state NMR analyses. The ^1^H and heteronuclear single quantum coherence (HSQC) NMR spectra (for indirect ^13^C detection) of the NCs exposed to OLPA revealed the emergence of two new distinct NMR peaks at 3.31 (with ^13^C at 51.9) and 3.11 (with ^13^C at 64.6) ppm (Figure 6d–f, h–g), whose intensity was observed to increase with the amount of OLPA added. The HSQC experiment, in the edited version, enabled to diphase the CH/CH_3_ with respect to CH_2_ and allowed to ascribe such new signals to CH_3_ and CH_2_ groups α to nitrogen group of DDA, respectively (Figure 6h). Notably, such new NMR peaks are shielded (*i*.*e*., at lower ppm) compared to those of surface-bound DDA and deshielded (*i*.*e*., at higher ppm) with respect to those of free DDABr molecules (Figure 6d–g). Moreover, such new NMR peaks are broader (fwhm = 24 Hz for the peak at 3.31 ppm; fwhm = 32 Hz for the peak at 3.11 ppm) with respect to those of free DDABr (fwhm = 6 Hz) and sharper compared to those of bound DDA (fwhm = 160 Hz). These results overall indicate that part of the DDA molecules is interacting with both the NCs’ surface and the electron-donating species. Such donating species are believed to be neutral OLPA only (acting as an L-type ligand), as also indicated by a control experiment in which the addition of OLPA to free DDABr molecules is observed to shift the diagnostic DDABr signals to lower ppm (Figure 6g).

To further elucidate the DDA–NC surface interaction, we performed 2D nuclear Overhauser effect spectroscopy (^1^H–^1^H NOESY) at 40 °C (see Figures 6i and S8).^25^ The NOESY evidences positive (blue) NOE cross peaks for OLPA signals (double bound region at 5.47 ppm) and negative (red) cross peaks for both the doublets of DDA (*i*.*e*., broad signals at 3.96 and 3.74 ppm and the new peaks at 3.31 end 3.11 ppm) (Figure 6i). These results indicate that OLPA molecules are free, while DDA species are in active dynamic binding with the surface of the NCs. On the basis of our NMR results, we conclude that OLPA molecules can interact with part of the DDA molecules present on the NC surface. Following this assumption, the integration of the NMR peaks in the 4.1–3.2 ppm range indicates that one-third of DDA ligands interact with OLPA when working with 3 or 10 equiv of OLPA.

To better understand the effects of the OLPA–DDA interaction, we cleaned the 3 equiv OLPA-treated NCs *via* the addition of ethyl acetate followed by centrifugation, and we performed the NMR analysis of both the supernatant and the precipitate (*i*.*e*., washed NCs) redispersed in toluene-*d*_8_ (Scheme 1). The NMR spectrum of the washed NCs indicated the absence of the peaks at 3.4 and 3.2 ppm (Figure 6c), which were instead detected in the supernatant (Figure S9). The ^31^P NMR analysis of the washed NCs also indicates the absence of surface-bound OLPA molecules, in agreement with the NOESY results (Figures S10 and 6i). Eventually, quantitative NMR analysis, carried out on solutions obtained by dissolving the washed NCs in DMSO-*d*,^39^ yielded a density of DDA ligands of 174 ligands/NC. Our NMR analysis, therefore, revealed that the treatment of DDABr-capped NCs with OLPA (and subsequent washing) leads to the removal of ∼40% of DDA surface ligands (the starting density was 291 ligands/NC), which corresponds to a reduction of DDA surface coverage from 42% to 25%. We would like to stress here that the density of DDA surface molecules cannot be reduced by simply further cleaning the starting DDABr-capped NCs with ethyl acetate; therefore the stripping observed here is ascribed to the treatment with OLPA. Moreover, as shown in Table S2, the XPS analysis further supported the stripping of DDA molecules, as the N amount decreased from 6.5% to 5.4% after the OLPA treatment, and proved that no etching occurred upon the stripping as the Cs/Pb/Br elemental ratio in the NCs remained unaltered. Notably, even after removing such a high fraction of DDA molecules, the optical properties of CsPbBr_3_ NCs were not much altered, with the PLQY being 78% after the removal of 40% of DDA molecules, experimentally proving the defect tolerance of such systems. Moreover, the NCs treated with OLPA exhibited a good structural stability when exposed to air up to 7 days, and the corresponding dispersions in hexane showed good optical stability when stored under an inert atmosphere up to 1 week (see Figures S11 and S12).

**Figure 7:**
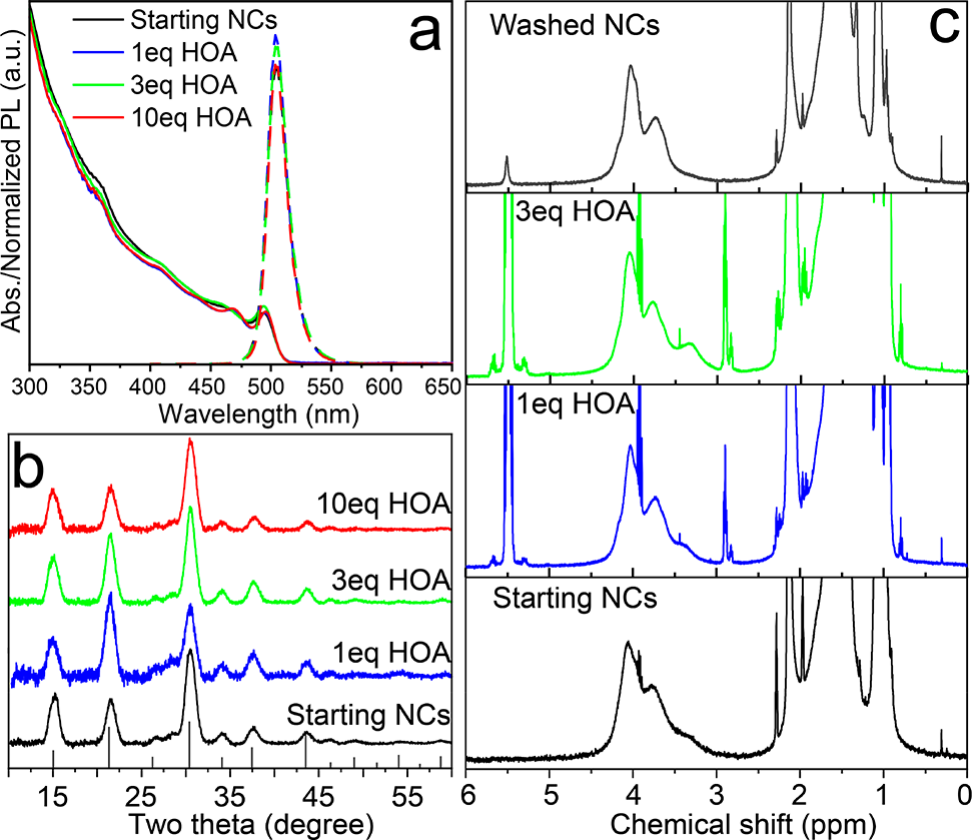
(a) Optical properties and (b) XRD patterns of DDABr-capped CsPbBr_3_ NCs treated with 1, 3, or 10 equiv of HOA. (c) ^1^H NMR spectra of DDABr-capped CsPbBr_3_, HOA treated, and washed (after the treatment with 3 equiv of HOA) NCs.

The treatment of the NCs with HOA led to similar results: overall, the optical properties, structure, and morphology of the final NCs were not affected by the addition of 1, 3, or even 10 equiv of HOA (see Figure 7a,b and Figure S13). Also in this case, the ^1^H NMR analysis indicated the emergence of a relatively broad NMR signal at ∼3.4 ppm, whose intensity increased together with the amount of added HOA (Figures 7c and S14) and which disappeared upon washing the NCs with EtAc (Figure 7c). In analogy with the OLPA case, these results suggest that HOA can bind and strip a fraction of DDA molecules from the surface of the NCs. From a rough and qualitative analysis of the NMR spectra, it is possible to ascertain that the addition of 3 equiv of HOA leads to the stripping of only a minimal quantity (∼2%) of surface DDA ligands (Figure 7c). After such stripping the PLQY was measured to be 79%.

On the other hand, DBSA was observed to interact with the NCs in a very different way. As shown in Figure S15, 1 equiv of DBSA quenched the PL emission of the NCs and led to their precipitation. The XRD analysis evidenced the absence of any Cs–Pb–Br phase, indicating the dissolution of the CsPbBr_3_ NCs (Figure S15). Hence DBSA, even in low amounts, etches the NCs, causing their dissolution and precipitation.

Overall, in agreement with the calculations, these results suggest that the higher the acidity of the ligands employed in the treatment, the higher the etching degree: while HOA (p*K*_a_ = 9.9) can strip a minor fraction of DDABr, OLPA (p*K*_a_ = 2) is able to remove up to 40% of surface DDABr. The acidity of DBSA (p*K*_a_ = −1.8), in turn, is possibly so high that it leads to a severe etching of the NCs.

## Conclusions

On the basis of the computational and experimental data of this work, we can draw some conclusions on the reactivity of exogenous acid/base ligands toward CsPbBr_3_ NCs:

(i) The process of ligand adsorption, *i*.*e*., a ligand binding the NC surface, although enthalpically favored, has an important entropic penalty. From our calculations, adsorption of L-type ligands is mostly prevented on {ABr} surfaces, but could take place on (PbBr_2_) ones, as also observed in a recent work.^35^

(ii) In the absence of native ligands that can accept protons, as in the case of DDABr-capped NCs, the chemisorption is highly unlikely, as it is always energetically unfavorable to extract the conjugate base of a strong acid. In other words, organic acids are not able to bind {ABr} or (PbBr_2_) surfaces *via* the displacement of Br^–^ ions (in the form of HBr).

(iii) The NC integrity can be undermined by etching, which occurs through the displacement of ABr or PbBr_2_ ion pairs operated by exogenous ligands. The etching process appears to be thermodynamically less costly for PbBr_2_ rather than ABr surfaces. In either case, the stronger the acid, the higher the etching degree. Indeed, experimentally we observed that oleic acid (p*K*_a_ = 9.9) can strip a minor fraction of DDABr, oleylphosphonic acid (p*K*_a_ = 2) can lead to the removal of up to 40% of DDA(Br), and dodecylbenzenesulfonic acid (p*K*_a_ = −1.8) completely etches the NCs. Despite the loss of ligand coverage, the emission characteristics of the NC remain unaltered, demonstrating the high surface tolerance of these materials. This ligand-stripping procedure also introduces a strategy to control the surface ligand coverage of perovskite NCs, which could be useful in optoelectronic devices, such as light-emitting diodes (where electrical resistance of the NC film should be minimized *via* ligand removal, while retaining high PL emission).

(iv) Basic ligands such as dioctylamine, trioctylphosphine, and trioctylphosphine oxide, which were computationally expected to displace PbBr_2_ units from the NCs, were experimentally observed to be inert even at high concentrations. In these cases, steric effects, not included in the calculations, are likely hindering any interaction between the ligands and the NCs. Of relevance is the case of octylamine, which, analogous to primary alkyl amines, has been widely reported to drive CsPbBr_3_ → Cs_4_PbBr_6_ (also called 3D → 0D) NC transformation. Our experimental data clearly show that neutral octylamine is not able to interact with the NCs when the system is completely aprotic (Figure S14). Interestingly, the same experiment if performed in completely anhydrous conditions, but employing a nondegassed octylamine, led to the 3D → 0D transformation (Figure S4). Our control experiments, thus, indicate that the presence of protons/moisture profoundly influences the ligands–NCs interaction when dealing with halide perovskite NCs.

## Methods

### Materials

Lead(II) acetate trihydrate (Pb(CH_3_COO)_2_·3H_2_O, 99.99%), cesium carbonate (Cs_2_CO_3_, 99%), benzoyl bromide (97%), 1-octadecene (ODE, 90%), anhydrous ethyl acetate (99.8%), anhydrous acetone, toluene (≥99.7%), deuterated toluene (toluene-*d*_8_, 99.8 at. % D), deuterated dimethyl sulfoxide (d-DMSO, 99.9 atom. % D), oleic acid (OA, 90%), didodecyldimethylammonium bromide (DDABr, 98%), 1-octylamine (99%), dioctylamine (98%), trioctylamine (98%), 1-octanal (99%), dioctyl sulfide (96%), 1-octanoic acid (%), 10-undecenoic acid (98%), diisooctylphosphinic acid (90%), 1-octylphosphonic acid (99%), 1-octanol (99%), and 1-octanethiol (98.5%) were purchased from Sigma. Didodecylamine (DDA, 97%) and 9-heptadecanone (98%) were purchased from TCI Chemicals. Trioctylphosphine (TOP, 97%) and trioctylphosphine oxide (99%, TOPO) were purchased from Strem Chemicals. All chemicals were used without further purification.

### Stock Solutions

For the Cs–Pb-oleate solution, Pb(CH_3_COO)_2_·3H_2_O (760 mg), Cs_2_CO_3_ (160 mg), and OA (15.00 mL) are loaded into a 25 mL three-neck flask and degassed on a Schlenk line (90 °C, 3 h) to form a solution containing Cs and Pb-oleates and to remove carbonic and acetic acids (byproducts). For the DDABr solution, DDABr (4.43 g) is dissolved in toluene (10.00 mL). For the benzoyl bromide solution, benzoyl bromide (1.00 mL) is dissolved in anhydrous toluene (10.00 mL). The Cs–Pb-oleate and the DDABr solutions are kept in air, while the benzoyl bromide is prepared and kept inside a nitrogen-filled glovebox.

### Synthesis of CsPbBr_3_ Nanocubes

The synthesis of CsPbBr_3_ NCs and subsequent ligand exchange reactions are performed following our previously reported methods with some modification.^25,37^ Briefly, the synthesis of starting NCs is performed in air, in a vial (20 mL) on a hot plate equipped with a thermocouple and a magnetic stirrer (1600 rpm). The Cs–Pb-oleate stock solution (1.50 mL) is loaded into a vial along with the DDA stock solution (1.50 mL) and 1-octadecene (9.00 mL). The mixture is heated to 70 °C, at which point the benzoyl bromide stock solution (0.55 mL) is injected. After 60 s, the reaction vial is cooled by plunging it into a water bath. The crude NC solution (3.00 mL) is then mixed with a toluene solution of DDAB (2 mL, 25 mM) and washed with ethyl acetate (20 mL). The NCs are separated by centrifugation at 6000 rpm, redispersed in a toluene solution of DDAB (1 mL, 2 mM), and washed a second time with ethyl acetate (6 mL). The NCs are again separated by centrifugation at 6000 rpm, redispersed in a toluene solution of DDAB (1 mL, 2 mM), and washed a third time with ethyl acetate (6 mL). Finally, the NCs are once more separated by centrifugation and redispersed in neat toluene.

### Reaction between Organic Ligands and DDA-Capped NCs

All organic ligands and solvent employed were anhydrous or degassed before using. Based on Method S3, the amount of exogenous ligands added was carefully calculated. The mixing process was operated in a glovebox filled with N_2_, and then the mixture was characterized by NMR without exposing the mixture to air/humidity. After the NMR characterization, the eventual NC washing was performed by using EtAc to remove the excess of added ligands and the DDA ligands stripped out.

### Inductively Coupled Plasma–Optical Emission Spectroscopy (ICP-OES)

We determine the concentration of NC dispersions in Pb by ICP-OES on an aiCAP 6000 spectrometer (Thermo Scientific). We use *aqua regia* to digest the NC solution overnight prior to the measurements.

### Nuclear Magnetic Resonance

NMR measurements are conducted at 300 K on a Bruker Avance III 400 MHz spectrometer equipped with a broad band inverse probe (BBI). Samples are prepared in deuterated toluene and loaded (*ca*. 700 μL) into 5 mm disposable sampleJet tubes. All ^1^H spectra are referred to the signal of residual nondeuterated solvent (calibrating at 7.09 and 2.50 ppm the toluene and DMSO signal, respectively), whereas the ^31^P spectra against an external reference solution of triethyl phosphate (10 mM) in d-toluene, calibrating the ^31^P signal at 0.00 ppm. ^1^H NMR spectra are acquired after an automatic 90° optimization routine performed on each sample tube,^45^ with 16–64 transients (depending on the sample concentration), 64k data points, no steady scan, and an interpulse delay of 30 s, over a spectral width of 20.55 ppm (offset at 6.18) and at a fixed receiver gain (1 for the concentrated free ligand samples and 65 for solutions of ligand-NCs). Spectra are manually phased and automatically baseline corrected. ^31^P spectra are acquired using an inverse gated ^1^H decoupled pulse sequence (Bruker libraries), using 1024–2048 transients (according to sample concentration), four steady scans, and an interpulse delay of 2 s, over a spectral width of 200.45 ppm (offset at 0.00 ppm). An apodization exponential function equivalent to 0.1 for ^1^H and 15 Hz (according to the S/N ratio) for ^31^P are applied to FIDs before Fourier transform.

To quantify the concentration of ligands, we evaporate the solvent from the NC dispersions under nitrogen flow and dissolve the solid residue in deuterated DMSO. We determine the concentration of ligands in the DMSO solution by comparing the integrated intensities of ligands’ ^1^H signals against that of a reference standard (maleic acid, 10 mM) measured independently following the PULCON (pulse length-based concentration determination) external standard procedure.^46^

### Steady-State UV–Vis Extinction Spectroscopy, Steady-State Photoluminescence Spectroscopy, and Photoluminescence Quantum Yields

We record optical extinction and photoluminescence spectra of dilute NC dispersions in toluene, inside quartz cuvettes with a 1 cm path-length, employing a Varian Cary 300 UV–vis spectrophotometer and a Varian Cary Eclipse fluorescence spectrophotometer, respectively. We measure absolute photoluminescence quantum yields using an Edinburgh Instruments FLS920 spectrofluorometer equipped with an integrating sphere, and the optical density of the NC solution was 0.15–0.20 at the excitation wavelength (400 nm). Time-correlated single photon counting (TCSPC) measurements are conducted in the same instrument using a pulsed 400 nm laser. TCSPC measurements are performed on concentrated NC dispersions, in a 45° geometry in order to minimize the self-absorption effects. The ligand-treated NCs are measured against an NC control (untreated DDA-capped NCs) set to the same optical density.

### Transmission Electron Microscopy (TEM)

NC dispersions are drop-cast on carbon-coated 200 mesh copper grids. We acquire bright-field TEM images on a JEOL JEM-1011 microscope (W filament) operating at an accelerating voltage of 100 kV.

### X-ray Photoelectron Spectroscopy (XPS)

The measurement was performed on a Kratos Axis UltraDLD spectrometer, equipped with a monochromatic Al Kα source, at 20 mA and 15 kV. Concentrated solutions of NCs were drop-cast onto freshly cleaved highly oriented pyrolytic graphite substrates. Survey scans were carried out using an analysis area of 300 × 700 μm and a pass energy of 160 eV. High-resolution scans were performed on the same analysis area, but with a pass energy of 10 eV. The Kratos charge neutralizer system was used on all specimens. Spectra were charge corrected to the main line of the carbon 1s spectrum (adventitious carbon) set to 284.8 eV. Spectra were analyzed using CasaXPS software (version 2.3.17).

### Computational Methodology

The CsPbBr_3_ nanocube models were encased by (100) facets, terminated by a PbBr_2_ inner shell, which in turn is capped by a CsBr outer shell (Figure 1a).^47^ This termination is consistent with the AX termination typically found in CsPbX_3_ NCs and with those synthesized in this work that have A = DDA, Cs and X = Br. Although it is not realistic for the NCs to be completely deprived of organic ligands, for the sake of our computations, describing the outer shell in its fully inorganic form is the only way to promote computational consistency and avoid effects that are difficult to estimate in the calculations. Furthermore, we are considering both CsBr and PbBr_2_ terminations in this study. The latter has been found in recent experimental works.^35,48^ In addition, in the case of CsBr-terminated crystals, the PbBr_2_ surface is accessible to exogenous ligands if CsBr surface vacancies are present.

Calculations are performed with the CP2K 6.1 package^49^ at the DFT/PBE level of theory^50^ with a double-ζ-type basis set (DZVP).^51^ Scalar relativistic effects are included in the calculations by means of effective core potentials, while spin–orbit coupling is neglected since its impact on the relaxed structure is negligible and calculations would be prohibitively demanding. Binding free energies of ligands on the surface of nanocrystals are computed as
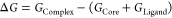
1where *G*_Complex_ is the total free energy of the complex (so, the free energy of the NC functionalized with a single ligand) in its ground state. *G*_Core_ is the free energy of the core of the NC. G_Ligand_ is the free energy of the isolated ligand, individually optimized. The free energy can be decomposed into terms of enthalpic Δ*H* and entropic Δ*S* contributions:
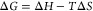
2where *T* is the temperature, which we will always consider at 298.15 K. For practical reasons, we can further decompose this expression as
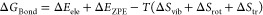
3where Δ*E*_ele_ is the electronic energy as obtained directly from the DFT calculations and Δ*E*_ZPE_ is the zero-point energy correction to the electronic energy. The expression of the entropy is based on the rigid-rotor harmonic oscillator approximation (RRHO)^52−54^ that allows to decouple rotational, translational, and vibrational contributions from each other. It must be noted that, due to the size of the NC model, no vibrational analysis is performed, so the definition of ground state, as referred to a global minimum of energy, must be more realistically considered as a local minimum of energy of the system. This is not expected to have a negative impact on the qualitative determination of the computed core–ligand bonding free energies; however this neglect entails that there is no change in the vibrational modes from the separated fragments to the supermolecular system. In other words, for all cases studied, the change in enthalpy is approximated as Δ*H* ∼ Δ*E*_el_, an approximation that is grounded also on the fact that ZPE correction is usually within a fraction of kcal/mol.^55^ The change in entropy can be regarded as Δ*S* ∼ (Δ*S*_rot_ + Δ*S*_tr_) and neglecting the vibrational term. This approximation of the entropy employed here is crude and is just meant to describe an upper bound correction to the free energy that tendentially favors separated fragments, if any. In the discussion in the main text, we provide a qualitative explanation of the role of entropy for each of the mechanisms studied. Details on the NC model and its size and stoichiometry are provided in the main text. Implicit solvent effects are also neglected because the low dielectric constants of commonly employed solvents in the experiment affect very little the energetics of binding.

## Supporting Information Available

Size histogram, determination of the NC stoichiometry, calculation of energetics of proton-induced ligand exchange and ligand-induced displacement, treatment of DDABr-capped NCs with degassed and nondegassed octylamine, absorption curves, TEM images, XRD patterns and NMR spectra of DDABr-capped NCs and several exogenous ligands (PDF)

## References

[ref1] Jena A. K.; Kulkarni A.; Miyasaka T. Halide Perovskite Photovoltaics: Background, Status, and Future Prospects. Chem. Rev. 2019, 119, 3036–3103. 10.1021/acs.chemrev.8b00539. 30821144

[ref2] Sutherland B. R.; Sargent E. H. Perovskite Photonic Sources. Nat. Photonics 2016, 10, 295 10.1038/nphoton.2016.62.

[ref3] Kovalenko M. V.; Protesescu L.; Bodnarchuk M. I. Properties and Potential Optoelectronic Applications of Lead Halide Perovskite Nanocrystals. Science 2017, 358, 745–750. 10.1126/science.aam7093. 29123061

[ref4] Quan L. N.; Rand B. P.; Friend R. H.; Mhaisalkar S. G.; Lee T.-W.; Sargent E. H. Perovskites for Next-Generation Optical Sources. Chem. Rev. 2019, 119, 7444–7477. 10.1021/acs.chemrev.9b00107. 31021609

[ref5] Yang D.; Li X.; Zeng H. Surface Chemistry of All Inorganic Halide Perovskite Nanocrystals: Passivation Mechanism and Stability. Adv. Mater. Interfaces 2018, 5, 1701662 10.1002/admi.201701662.

[ref6] Shamsi J.; Urban A. S.; Imran M.; De Trizio L.; Manna L. Metal Halide Perovskite Nanocrystals: Synthesis, Post-Synthesis Modifications, and Their Optical Properties. Chem. Rev. 2019, 119, 3296–3348. 10.1021/acs.chemrev.8b00644. 30758194 PMC6418875

[ref7] Akkerman Q. A.; Rainò G.; Kovalenko M. V.; Manna L. Genesis, Challenges and Opportunities for Colloidal Lead Halide Perovskite Nanocrystals. Nat. Mater. 2018, 17, 394–405. 10.1038/s41563-018-0018-4. 29459748

[ref8] Protesescu L.; Yakunin S.; Bodnarchuk M. I.; Krieg F.; Caputo R.; Hendon C. H.; Yang R. X.; Walsh A.; Kovalenko M. V. Nanocrystals of Cesium Lead Halide Perovskites (CsPbX_3_, X = Cl, Br, and I): Novel Optoelectronic Materials Showing Bright Emission with Wide Color Gamut. Nano Lett. 2015, 15, 3692–3696. 10.1021/nl5048779. 25633588 PMC4462997

[ref9] Imran M.; Caligiuri V.; Wang M.; Goldoni L.; Prato M.; Krahne R.; De Trizio L.; Manna L. Benzoyl Halides as Alternative Precursors for the Colloidal Synthesis of Lead-Based Halide Perovskite Nanocrystals. J. Am. Chem. Soc. 2018, 140, 2656–2664. 10.1021/jacs.7b13477. 29378131 PMC5908184

[ref10] Liu X.-K.; Xu W.; Bai S.; Jin Y.; Wang J.; Friend R. H.; Gao F. Metal Halide Perovskites for Light-Emitting Diodes. Nat. Mater. 2021, 20, 10–21. 10.1038/s41563-020-0784-7. 32929252

[ref11] Gandini M.; Villa I.; Beretta M.; Gotti C.; Imran M.; Carulli F.; Fantuzzi E.; Sassi M.; Zaffalon M.; Brofferio C.; Manna L.; Beverina L.; Vedda A.; Fasoli M.; Gironi L.; Brovelli S. Efficient, Fast and Reabsorption-Free Perovskite Nanocrystal-Based Sensitized Plastic Scintillators. Nat. Nanotechnol. 2020, 15, 462–468. 10.1038/s41565-020-0683-8. 32424340

[ref12] Fu Y.; Zhu H.; Chen J.; Hautzinger M. P.; Zhu X. Y.; Jin S. Metal Halide Perovskite Nanostructures for Optoelectronic Applications and the Study of Physical Properties. Nat. Rev. Mater. 2019, 4, 169–188. 10.1038/s41578-019-0080-9.

[ref13] Song J.; Li J.; Li X.; Xu L.; Dong Y.; Zeng H. Quantum Dot Light-Emitting Diodes Based on Inorganic Perovskite Cesium Lead Halides (CsPbX_3_). Adv. Mater. 2015, 27, 7162–7167. 10.1002/adma.201502567. 26444873

[ref14] Swarnkar A.; Chulliyil R.; Ravi V. K.; Irfanullah M.; Chowdhury A.; Nag A. Colloidal CsPbBr_3_ Perovskite Nanocrystals: Luminescence beyond Traditional Quantum Dots. Angew. Chem. Int. Ed. Engl. 2015, 54, 15424–15428. 10.1002/anie.201508276. 26546495

[ref15] Zheng X.; Hou Y.; Sun H.-T.; Mohammed O. F.; Sargent E. H.; Bakr O. M. Reducing Defects in Halide Perovskite Nanocrystals for Light-Emitting Applications. J. Phys. Chem. Lett. 2019, 10, 2629–2640. 10.1021/acs.jpclett.9b00689. 31038960

[ref16] ten Brinck S.; Zaccaria F.; Infante I. Defects in Lead Halide Perovskite Nanocrystals: Analogies and (Many) Differences with the Bulk. ACS Energy Lett. 2019, 4, 2739–2747. 10.1021/acsenergylett.9b01945.

[ref17] Almeida G.; Infante I.; Manna L. Resurfacing Halide Perovskite Nanocrystals. Science 2019, 364, 833–834. 10.1126/science.aax5825. 31147510

[ref18] Zito J.; Infante I. The Future of Ligand Engineering in Colloidal Semiconductor Nanocrystals. Acc. Chem. Res. 2021, 54, 1555–1564. 10.1021/acs.accounts.0c00765. 33635646 PMC8028043

[ref19] Smock S. R.; Chen Y.; Rossini A. J.; Brutchey R. L. The Surface Chemistry and Structure of Colloidal Lead Halide Perovskite Nanocrystals. Acc. Chem. Res. 2021, 54, 707–718. 10.1021/acs.accounts.0c00741. 33449626

[ref20] Xue J.; Wang R.; Yang Y. The Surface of Halide Perovskites from Nano to Bulk. Nat. Rev. Mater. 2020, 5, 809–827. 10.1038/s41578-020-0221-1.

[ref21] Qiao T.; Son D. H. Synthesis and Properties of Strongly Quantum-Confined Cesium Lead Halide Perovskite Nanocrystals. Acc. Chem. Res. 2021, 54, 1399–1408. 10.1021/acs.accounts.0c00706. 33566565

[ref22] De Roo J.; Ibáñez M.; Geiregat P.; Nedelcu G.; Walravens W.; Maes J.; Martins J. C.; Van Driessche I.; Kovalenko M. V.; Hens Z. Highly Dynamic Ligand Binding and Light Absorption Coefficient of Cesium Lead Bromide Perovskite Nanocrystals. ACS Nano 2016, 10, 2071–2081. 10.1021/acsnano.5b06295. 26786064

[ref23] Chen Y.; Smock S. R.; Flintgruber A. H.; Perras F. A.; Brutchey R. L.; Rossini A. J. Surface Termination of CsPbBr_3_ Perovskite Quantum Dots Determined by Solid-State Nmr Spectroscopy. J. Am. Chem. Soc. 2020, 142, 6117–6127. 10.1021/jacs.9b13396. 32115949

[ref24] Smock S. R.; Williams T. J.; Brutchey R. L. Quantifying the Thermodynamics of Ligand Binding to CsPbBr_3_ Quantum Dots. Angew. Chem. Int. Ed. Engl. 2018, 57, 11711–11715. 10.1002/anie.201806916. 30051545 PMC6467082

[ref25] Imran M.; Ijaz P.; Goldoni L.; Maggioni D.; Petralanda U.; Prato M.; Almeida G.; Infante I.; Manna L. Simultaneous Cationic and Anionic Ligand Exchange for Colloidally Stable CsPbBr_3_ Nanocrystals. ACS Energy Lett. 2019, 4, 819–824. 10.1021/acsenergylett.9b00140.

[ref26] Bodnarchuk M. I.; Boehme S. C.; ten Brinck S.; Bernasconi C.; Shynkarenko Y.; Krieg F.; Widmer R.; Aeschlimann B.; Günther D.; Kovalenko M. V.; Infante I. Rationalizing and Controlling the Surface Structure and Electronic Passivation of Cesium Lead Halide Nanocrystals. ACS Energy Lett. 2019, 4, 63–74. 10.1021/acsenergylett.8b01669. 30662955 PMC6333230

[ref27] Koscher B. A.; Swabeck J. K.; Bronstein N. D.; Alivisatos A. P. Essentially Trap-Free CsPbBr_3_ Colloidal Nanocrystals by Postsynthetic Thiocyanate Surface Treatment. J. Am. Chem. Soc. 2017, 139, 6566–6569. 10.1021/jacs.7b02817. 28448140

[ref28] Ahmed T.; Seth S.; Samanta A. Boosting the Photoluminescence of CsPbX_3_ (X = Cl, Br, I) Perovskite Nanocrystals Covering a Wide Wavelength Range by Postsynthetic Treatment with Tetrafluoroborate Salts. Chem. Mater. 2018, 30, 3633–3637. 10.1021/acs.chemmater.8b01235.

[ref29] Wu Y.; Wei C.; Li X.; Li Y.; Qiu S.; Shen W.; Cai B.; Sun Z.; Yang D.; Deng Z.; Zeng H. *In Situ* Passivation of Pbbr64– Octahedra toward Blue Luminescent CsPbBr_3_ Nanoplatelets with near 100% Absolute Quantum Yield. ACS Energy Lett. 2018, 3, 2030–2037. 10.1021/acsenergylett.8b01025.

[ref30] Liu Z.; Bekenstein Y.; Ye X.; Nguyen S. C.; Swabeck J.; Zhang D.; Lee S.-T.; Yang P.; Ma W.; Alivisatos A. P. Ligand Mediated Transformation of Cesium Lead Bromide Perovskite Nanocrystals to Lead Depleted Cs_4_PbBr_6_ Nanocrystals. J. Am. Chem. Soc. 2017, 139, 5309–5312. 10.1021/jacs.7b01409. 28358191

[ref31] Almeida G.; Goldoni L.; Akkerman Q.; Dang Z.; Khan A. H.; Marras S.; Moreels I.; Manna L. Role of Acid–Base Equilibria in the Size, Shape, and Phase Control of Cesium Lead Bromide Nanocrystals. ACS Nano 2018, 12, 1704–1711. 10.1021/acsnano.7b08357. 29381326 PMC5830690

[ref32] Udayabhaskararao T.; Houben L.; Cohen H.; Menahem M.; Pinkas I.; Avram L.; Wolf T.; Teitelboim A.; Leskes M.; Yaffe O.; Oron D.; Kazes M. A Mechanistic Study of Phase Transformation in Perovskite Nanocrystals Driven by Ligand Passivation. Chem. Mater. 2018, 30, 84–93. 10.1021/acs.chemmater.7b02425.

[ref33] De Roo J.; De Keukeleere K.; Hens Z.; Van Driessche I. From Ligands to Binding Motifs and beyond; The Enhanced Versatility of Nanocrystal Surfaces. Dalton Trans. 2016, 45, 13277–13283. 10.1039/C6DT02410F. 27461488

[ref34] Zhou Y.; Wang F.; Buhro W. E. Large Exciton Energy Shifts by Reversible Surface Exchange in 2D II–VI Nanocrystals. J. Am. Chem. Soc. 2015, 137, 15198–15208. 10.1021/jacs.5b09343. 26568026

[ref35] Zhang B.; Goldoni L.; Lambruschini C.; Moni L.; Imran M.; Pianetti A.; Pinchetti V.; Brovelli S.; De Trizio L.; Manna L. Stable and Size Tunable CsPbBr_3_ Nanocrystals Synthesized with Oleylphosphonic Acid. Nano Lett. 2020, 20, 8847–8853. 10.1021/acs.nanolett.0c03833. 33201718 PMC7872419

[ref36] Quarta D.; Imran M.; Capodilupo A.-L.; Petralanda U.; van Beek B.; De Angelis F.; Manna L.; Infante I.; De Trizio L.; Giansante C. Stable Ligand Coordination at the Surface of Colloidal CsPbBr_3_ Nanocrystals. J. Phys. Chem. Lett. 2019, 10, 3715–3726. 10.1021/acs.jpclett.9b01634. 31244273

[ref37] Imran M.; Ijaz P.; Baranov D.; Goldoni L.; Petralanda U.; Akkerman Q.; Abdelhady A. L.; Prato M.; Bianchini P.; Infante I.; Manna L. Shape-Pure, Nearly Monodispersed CsPbBr_3_ Nanocubes Prepared Using Secondary Aliphatic Amines. Nano Lett. 2018, 18, 7822–7831. 10.1021/acs.nanolett.8b03598. 30383965 PMC6428374

[ref38] Hens Z.; Martins J. C. A Solution Nmr Toolbox for Characterizing the Surface Chemistry of Colloidal Nanocrystals. Chem. Mater. 2013, 25, 1211–1221. 10.1021/cm303361s.

[ref39] Zhang B.; Wang M.; Ghini M.; Melcherts A. E. M.; Zito J.; Goldoni L.; Infante I.; Guizzardi M.; Scotognella F.; Kriegel I.; De Trizio L.; Manna L. Colloidal Bi-Doped Cs_2_Ag_1–x_Na_x_InCl_6_ Nanocrystals: Undercoordinated Surface Cl Ions Limit Their Light Emission Efficiency. ACS Mater. Lett. 2020, 2, 1442–1449. 10.1021/acsmaterialslett.0c00359. 33644762 PMC7901666

[ref40] Toso S.; Baranov D.; Manna L. Hidden in Plain Sight: The Overlooked Influence of the Cs^+^ Substructure on Transformations in Cesium Lead Halide Nanocrystals. ACS Energy Lett. 2020, 5, 3409–3414. 10.1021/acsenergylett.0c02029.

[ref41] Leemans J.; Dümbgen K. C.; Minjauw M. M.; Zhao Q.; Vantomme A.; Infante I.; Detavernier C.; Hens Z. Acid–Base Mediated Ligand Exchange on Near-Infrared Absorbing, Indium-Based III–V Colloidal Quantum Dots. J. Am. Chem. Soc. 2021, 143, 4290–4301. 10.1021/jacs.0c12871. 33710882

[ref42] Palazon F.; Almeida G.; Akkerman Q. A.; De Trizio L.; Dang Z.; Prato M.; Manna L. Changing the Dimensionality of Cesium Lead Bromide Nanocrystals by Reversible Postsynthesis Transformations with Amines. Chem. Mater. 2017, 29, 4167–4171. 10.1021/acs.chemmater.7b00895. 28572702 PMC5445717

[ref43] Toso S.; Baranov D.; Altamura D.; Scattarella F.; Dahl J.; Wang X.; Marras S.; Alivisatos A. P.; Singer A.; Giannini C.; Manna L. Multilayer Diffraction Reveals That Colloidal Superlattices Approach the Structural Perfection of Single Crystals. ACS Nano 2021, 15, 6243–6256. 10.1021/acsnano.0c08929. 33481560 PMC8155329

[ref44] Toso S.; Baranov D.; Giannini C.; Marras S.; Manna L. Wide-Angle X-Ray Diffraction Evidence of Structural Coherence in CsPbBr_3_ Nanocrystal Superlattices. ACS Mater. Lett. 2019, 1, 272–276. 10.1021/acsmaterialslett.9b00217. 32954357 PMC7497715

[ref45] Wu P. S. C.; Otting G. Rapid Pulse Length Determination in High-Resolution Nmr. J. Magn. Reson. 2005, 176, 115–119. 10.1016/j.jmr.2005.05.018. 15972263

[ref46] Wider G.; Dreier L. Measuring Protein Concentrations by Nmr Spectroscopy. J. Am. Chem. Soc. 2006, 128, 2571–2576. 10.1021/ja055336t. 16492040

[ref47] ten Brinck S.; Infante I. Surface Termination, Morphology, and Bright Photoluminescence of Cesium Lead Halide Perovskite Nanocrystals. ACS Energy Lett. 2016, 1, 1266–1272. 10.1021/acsenergylett.6b00595.

[ref48] Zhang B.; Goldoni L.; Zito J.; Dang Z.; Almeida G.; Zaccaria F.; de Wit J.; Infante I.; De Trizio L.; Manna L. Alkyl Phosphonic Acids Deliver CsPbBr_3_ Nanocrystals with High Photoluminescence Quantum Yield and Truncated Octahedron Shape. Chem. Mater. 2019, 31, 9140–9147. 10.1021/acs.chemmater.9b03529.

[ref49] Kühne T. D.; Iannuzzi M.; Ben M. D.; Rybkin V. V.; Seewald P.; Stein F.; Laino T.; Khaliullin R. Z.; Schütt O.; Schiffmann F.; Golze D.; Wilhelm J.; Chulkov S.; Bani-Hashemian M. H.; Weber V.; Borštnik U.; Taillefumier M.; Jakobovits A. S.; Lazzaro A.; Pabst H.; et al. cp2k: An Electronic Structure and Molecular Dynamics Software Package - Quickstep: Efficient and Accurate Electronic Structure Calculations. J. Chem. Phys. 2020, 152, 194103 10.1063/5.0007045. 33687235

[ref50] Perdew J. P.; Burke K.; Ernzerhof M. Generalized Gradient Approximation Made Simple. Phys. Rev. Lett. 1996, 77, 3865–3868. 10.1103/PhysRevLett.77.3865. 10062328

[ref51] VandeVondele J.; Hutter J. Gaussian Basis Sets for Accurate Calculations on Molecular Systems in Gas and Condensed Phases. J. Chem. Phys. 2007, 127, 114105 10.1063/1.2770708. 17887826

[ref52] Pople J. A.; Schlegel H. B.; Krishnan R.; Defrees D. J.; Binkley J. S.; Frisch M. J.; Whiteside R. A.; Hout R. F.; Hehre W. J. Molecular Orbital Studies of Vibrational Frequencies. Int. J. Quantum Chem. 1981, 20, 269–278. 10.1002/qua.560200829.

[ref53] Stratmann R. E.; Burant J. C.; Scuseria G. E.; Frisch M. J. Improving Harmonic Vibrational Frequencies Calculations in Density Functional Theory. J. Chem. Phys. 1997, 106, 10175–10183. 10.1063/1.474047.

[ref54] Fogarasi G.; Pulay P. *Ab Initio* Vibrational Force Fields. Annu. Rev. Phys. Chem. 1984, 35, 191–213. 10.1146/annurev.pc.35.100184.001203.

[ref55] Procacci P. Reformulating the Entropic Contribution in Molecular Docking Scoring Functions. J. Comput. Chem. 2016, 37, 1819–1827. 10.1002/jcc.24397. 27231844

