# Supplementary material for: The Reactivity of CsPbBr3 Nanocrystals toward Acid/Base Ligands
Source: ACS Nano. 2022 Jan 10;16(1):1444–55. doi: 10.1021/acsnano.1c09603 (PMC8793808; doi:10.1021/acsnano.1c09603)
Supplement: Supplementary file 1 — nn1c09603_si_001.pdf [file nn1c09603_si_001.pdf]

Supporting Information for:

The Reactivity of $CsPbBr_3$ Nanocrystals towards Acid/Base Ligands

Francesco Zaccaria,[a‡] Baowei Zhang,[a,b‡], Luca Goldoni,[c] Muhammad Imran,[a] Juliette Zito,[a,b] Bas van Beek,[e] Simone Lauciello,[d] Luca De Trizio,[a*] Liberato Manna,[a*] and Ivan Infante[a,e*]

[a]Department of Nanochemistry, [c]Analytical Chemistry Lab and [d]Electron Microscopy Facility, Istituto Italiano di Tecnologia, Via Morego 30, 16163 Genova, Italy

[b]Dipartimento di Chimica e Chimica Industriale, Università degli Studi di Genova, Via Dodecaneso 31, 16146 Genova, Italy.

[e]Department of Theoretical Chemistry, Faculty of Science, Vrije Universiteit Amsterdam, de Boelelaan 1083, 1081 HV Amsterdam, The Netherlands

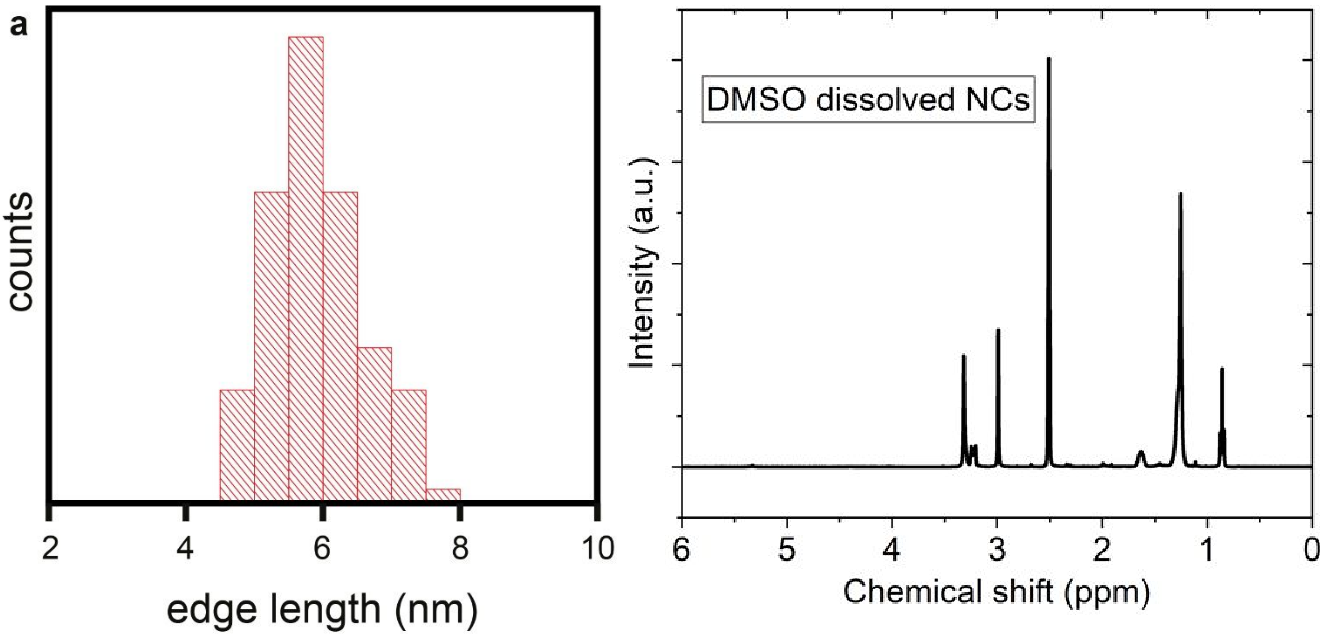

Figure S1. a) Size histogram of DDABr-capped nanocrystals obtained *via* analysis of TEM images; b) ^{1}H-NMR of DMSO dissolved DDAB caped NCs.

Method S1. Determination of the NC stoichiometry, concentration and absorption coefficient

According to the composition obtained from EDS ($Cs_{1.0}Pb_{1.0}Br_{2.9}$), the DDA-capped $CsPbBr_3$ NCs appear to be terminated by a $PbBr_2$ inner shell and (partially) capped by a hybrid AX outer shell (A=Cs, DDA; X=Br, oleate). We assume that such nanocubes can be approximated to a charge balanced CsBr-capped $CsPbBr_3$ NC with an edge length of 6.4 nm containing 1694 Cs atoms, 1331 Pb atoms and 4356 Br atoms if fully capped (see Table S1).

Starting from this model, we determined the concentration of NCs in solution by measuring the concentration of Pb ions *via* inductively coupled plasma - optical emission spectrometry (ICP-OES), and dividing it for 1331 (Pb atoms per NC). Eventually, we measured the concentration of ligands (DDA, oleate) in the dispersions using quantitative NMR: we dissolved the NCs in DMSO and performed a quantitative NMR analysis, as shown in Figure S1b, which yielded a surface concentration of 291 DDA^+ molecules per NC (and 21 oleate species per NC).

These analyses allowed us to reveal the composition of DDABr-capped $CsPbBr_3$ NCs. Results are summarized in Table S1. Assuming that all ligands quantified by NMR are bound (indeed we did not find ^1H peaks ascribable to free OA or DDABr), the outer shell result to have a composition of $(Cs_{0.48}DDA_{0.42})(Br_{0.32}Oleate_{0.03})$. The evident Br-deficiency is most likely due to Br desorption under SEM-EDS analysis conditions. In order to maintain charge balance the actual Br content in the outer shell is expected to be around 87%. We therefore describe the NCs as:

$[CsPbBr_3](PbBr_2)\{(Cs_{0.48}DDA_{0.42})(Br_{0.87}Oleate_{0.03})(o)_{0.10}\}$

In the formula above, we define (o) as AX surface vacancies. This overall composition corresponds to a surface (outer shell) coverage (occupation) of 90%.

Table S1 – Composition of the DDA-capped $CsPbBr_3$ nanocrystals assuming that the total number of sites is that of a CsBr capped $CsPbBr_3$ NCs with an edge length of 6.4 nm

Site	Specie	Number of sites			
		Total	Core	Inner-shell	Outer shell
**A**	**total**	1694	1000	-	694
	Cs	1331	1000	-	331
	DDA	290	-	-	290
	Vac.	73	-	-	73
**M**	**total**	1331	729	602	-
	Pb	1331	729	602	-
**X**	**total**	4356	2430	1200	726
	Br	3860	2430	1200	230
	Oleate	20	-	-	20
	Vac.	476	-	-	476

Method S2. Surface Reactivity

Following the nomenclature introduced in the main text of the manuscript, the *adsorption* of a ligand L onto the ABr outer shell of the NCs (Figure 2a, left side) can be written as:

$[CsPbBr_3](PbBr_2)_k\{ABr\}_m + L \rightarrow [CsPbBr_3](PbBr_2)_k\{ABr\}_{m-1}\{ABr\text{-}L\}$ **(Eq. 1)**

where the added ligand will passivate a single {ABr} surface site. Accordingly, considering a fully de-capped outer-shell, the adsorption on the $PbBr_2$ shell can be written as (Figure 2a, right side):

$[CsPbBr_3](PbBr_2)_k + L \rightarrow [CsPbBr_3](PbBr_2)_{k-1}(PbBr_2\text{-}L)$ **(Eq.2)**

Protic ligands (HL) may also *chemisorb* onto the NCs' surface by donating their protons to surface anions (Figure 2b), and therefore chemisorption may lead only to the formation of HBr. If this process occurs on an ABr surface, it can be written as

$[CsPbBr_3](PbBr_2)_k\{ABr\}_m + HL \rightarrow [CsPbBr_3](PbBr_2)_k\{ABr\}_{m-1}\{ABr\text{-}HL\} \rightarrow$
$[CsPbBr_3](PbBr_2)_k\{ABr\}_{m-1}\{AL\} + HBr$ **(Eq. 3)**

If instead it occurs on a $PbBr_2$ surface, it can be written as

$[CsPbBr_3](PbBr_2)_k + HL \rightarrow [CsPbBr_3](PbBr_{2k-1}(PbBr_2\text{-}HL) \rightarrow [CsPbBr_3](PbBr_2)_{k-1}(PbBr_2\text{-}L) + HBr$
(Eq. 4)

Finally, we also consider a more complex *etching* reaction in which the ligands remove portions of the original NCs. In particular we consider the removal of ABr or $PbBr_2$ units from the NCs (Figure 2c):[15]

$[CsPbBr_3](PbBr_2)_k\{ABr\}_m + nL \rightarrow [CsPbBr_3](PbBr_2)_k\{ABr\}_{m-1}\{o\} + AX\text{-}L_n$ **(Eq. 5)**

$[CsPbBr_3](PbBr_2)_k + nL \rightarrow [CsPbBr_3](PbBr_2)_{k-1}(o) + PbBr_2\text{-}L_n$ **(Eq. 6)**

where we introduced the notation {o} and (o) to indicate a surface vacancy at the outer and/or inner shells, respectively.

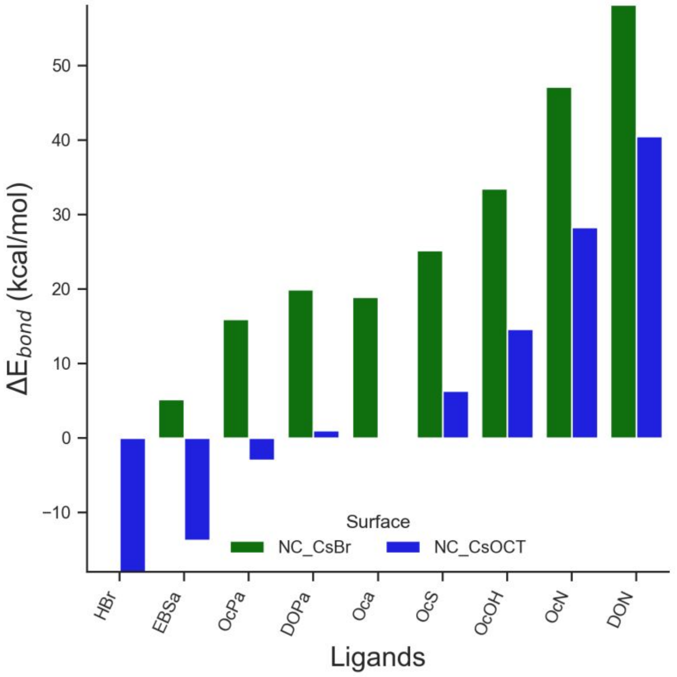

Figure S2. Energetics of proton-induced ligand exchange of bromide ions (green bars) and octanoate (blue bars) with a series of ligands of various acidity. Unlike bromide, octanoate can be displaced by acids stronger than the octanoic such as halic, sulphonic and phosphonic acids (negative values of blue bars).

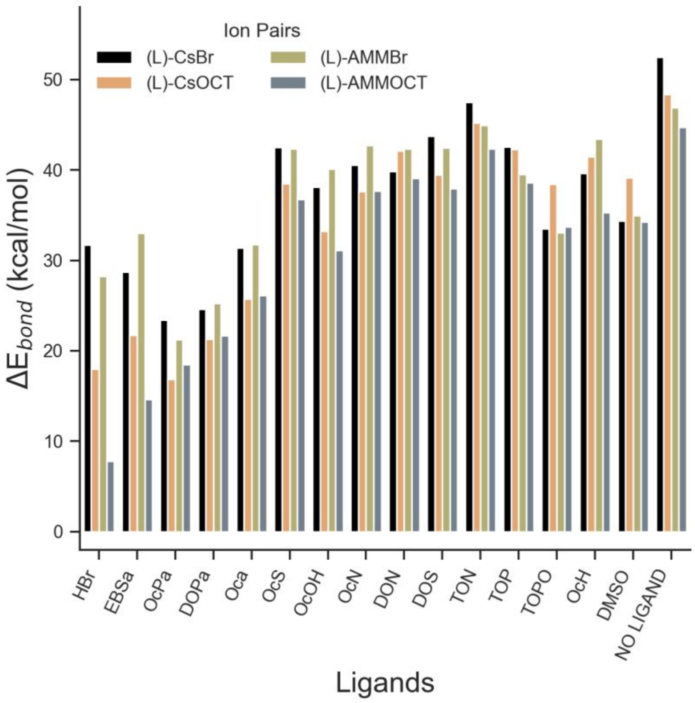

Figure S3. Ligand-induced displacement of the most commonly used passivating ligand pairs from the $CsPbBr_3$ NC surface. Caesium octanoate, ammonium bromide and ammonium octanoate are found to be bound to the surface with energies of the same order of magnitude of CsBr with only minor differences.

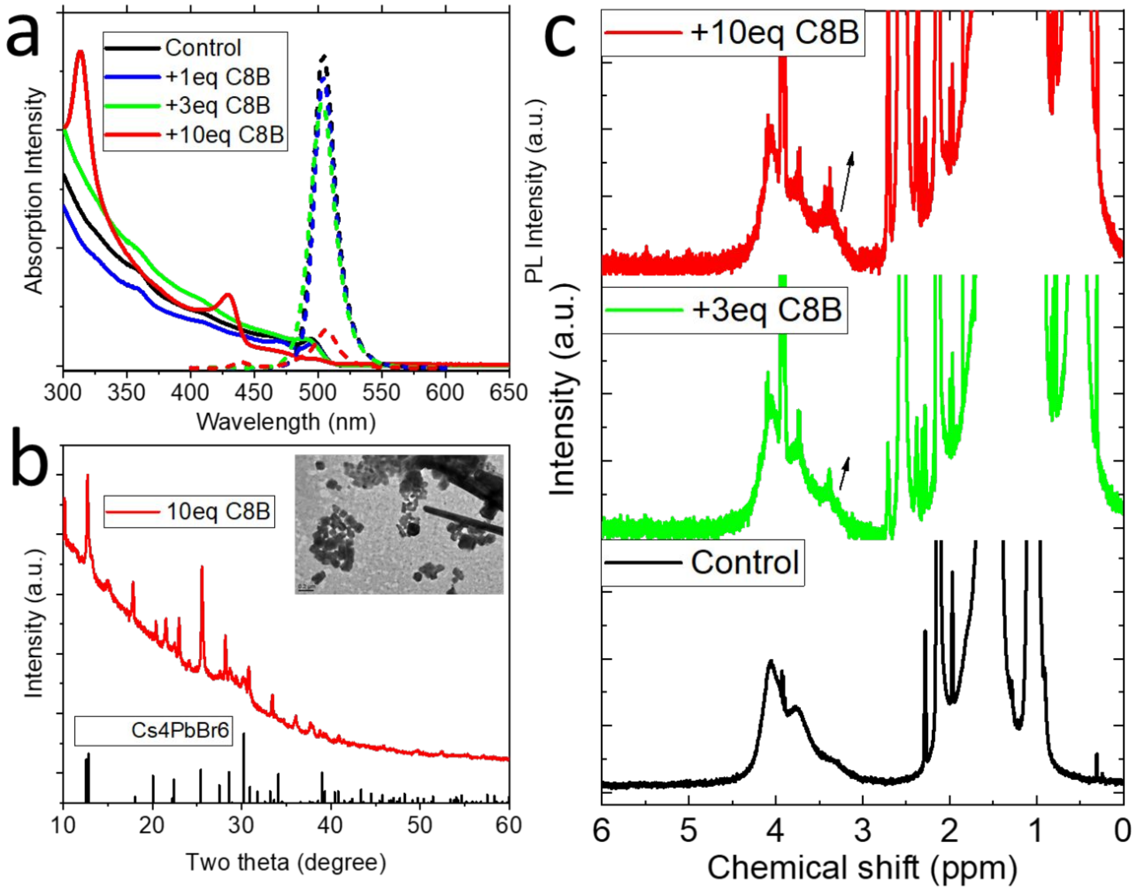

Figure S4. DDABr-capped $CsPbBr_3$ nanocrystals treated with 1-10 eq amount of non-degassed octylamine (C8B) and the corresponding (a) Absorption and PL intensity curves; (b) XRD pattern and (inset) TEM image; (c) ^1H NMR spectra. It is possible to notice here that non-degassed C8B is able to transform $CsPbBr_3$ NCs into Cs_4PbBr_6 (panel b), and to interact with the surface DDA molecules (panel c, black arrows).

Method S3. Addition of exogenous ligands.

The concentration of [NCs] in a given NC dispersion in toluene was determined by measuring the absorbance of the latter at $\lambda = 400$ nm and by employing the calibration curve reported by Jorick Maes *et al.* [J. Phys. Chem. Lett. 2018, 9, 3093−3097]. Typical [NCs] were in the 16-38 uM order.

Considering a concentration of 38 uM the equivalents of added exogenous ligands were calculated as follow:

$N_{Surface\ site} = 726$ per NCs; $N_A = 6.02*10^{23}$

1 eq ligands = $[NCs]\times N_A \times N_{Surface\ site} \times 1 = 5.7$ uM

3 eq ligands = $[NCs] \times N_A \times N_{Surface\ site} \times 3 = 17.1$ uM

10 eq ligands = $[NCs] \times N_A \times N_{Surface\ site} \times 10 = 57$ uM

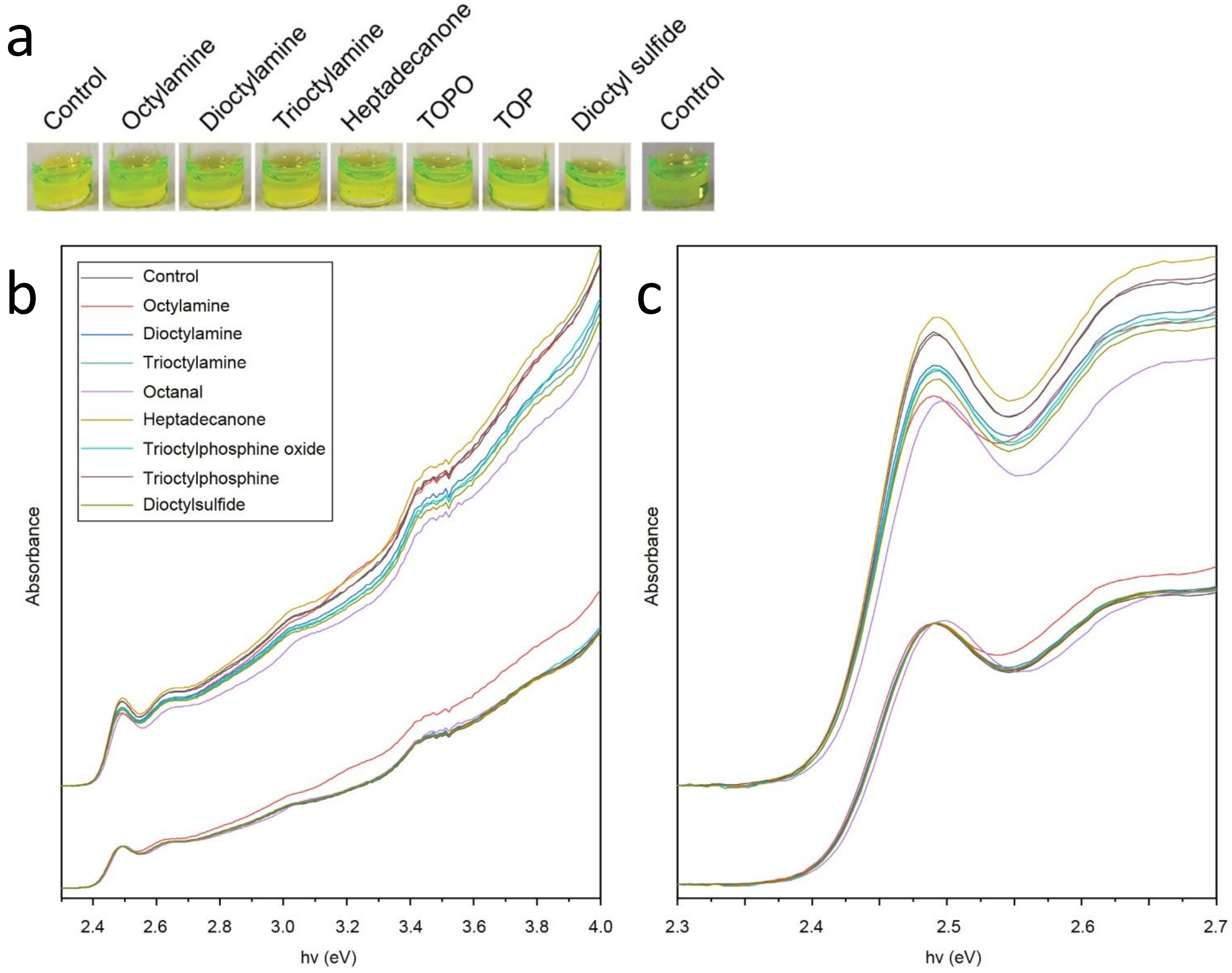

Figure S5. Photographs (a) and (b-c) absorbance spectra of samples treated with basic ligands at a concentration of 10 ligands per surface site and diluted for analysis. Background (2.3 eV) subtracted spectra are shown on top, while spectra normalized at 2.489 eV are shown below.

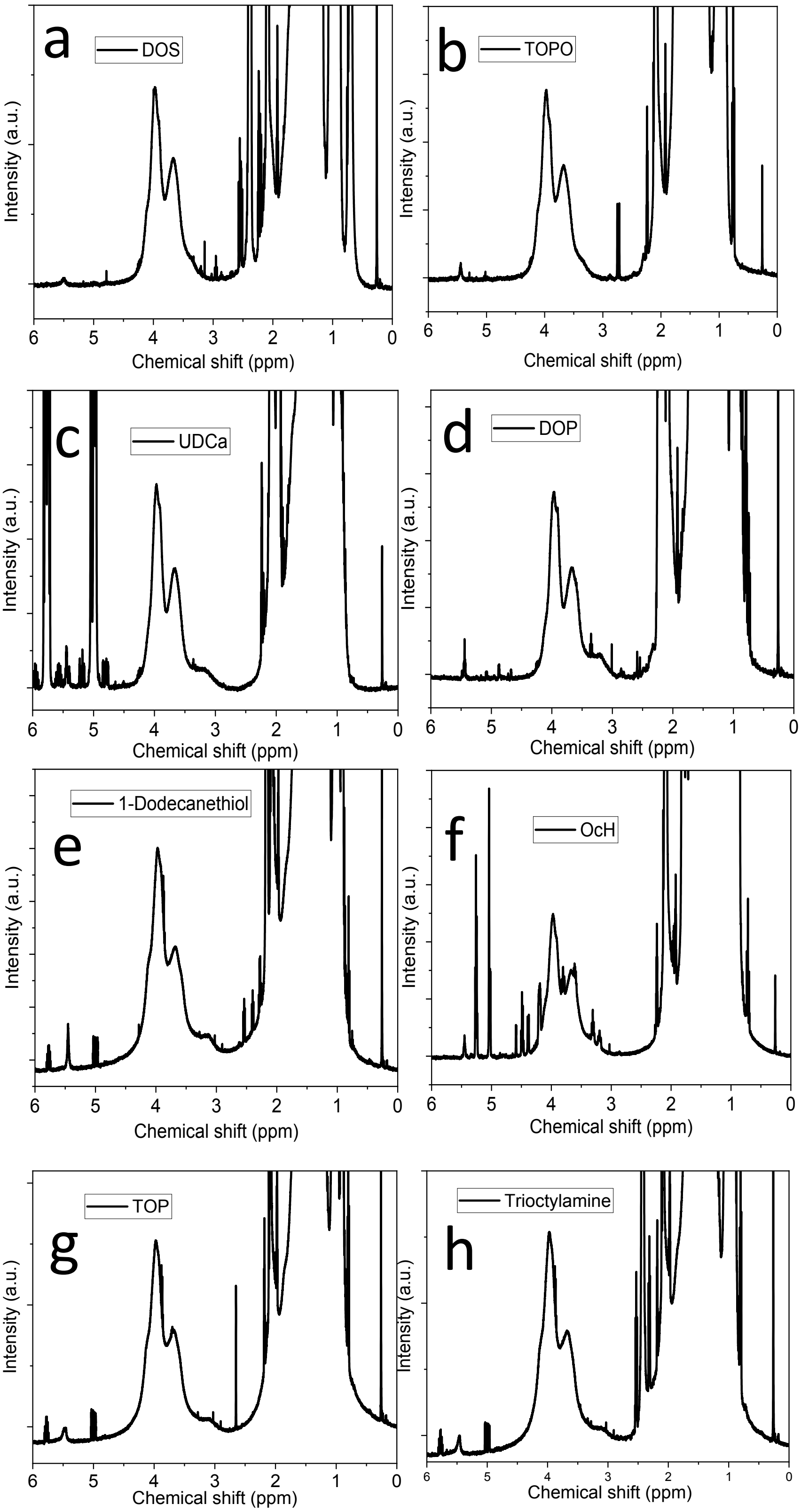

Figure S6. 1H-NMR spectra of DDAB NCs treated by various excess (10eq) neutral ligands: (a) Dioctylsulfide (Dos); (b) Trioctylphospine oxide (TOPO); (c) Undecanoic acid (UDCa); (d) Dioctylphosphinic acid (DOpa); (e) 1-Dodecanethiol (OcN); (f) Octanal (OcH); (g) trioctylphosphine (TOP); (h) Trioctylamine (TON).

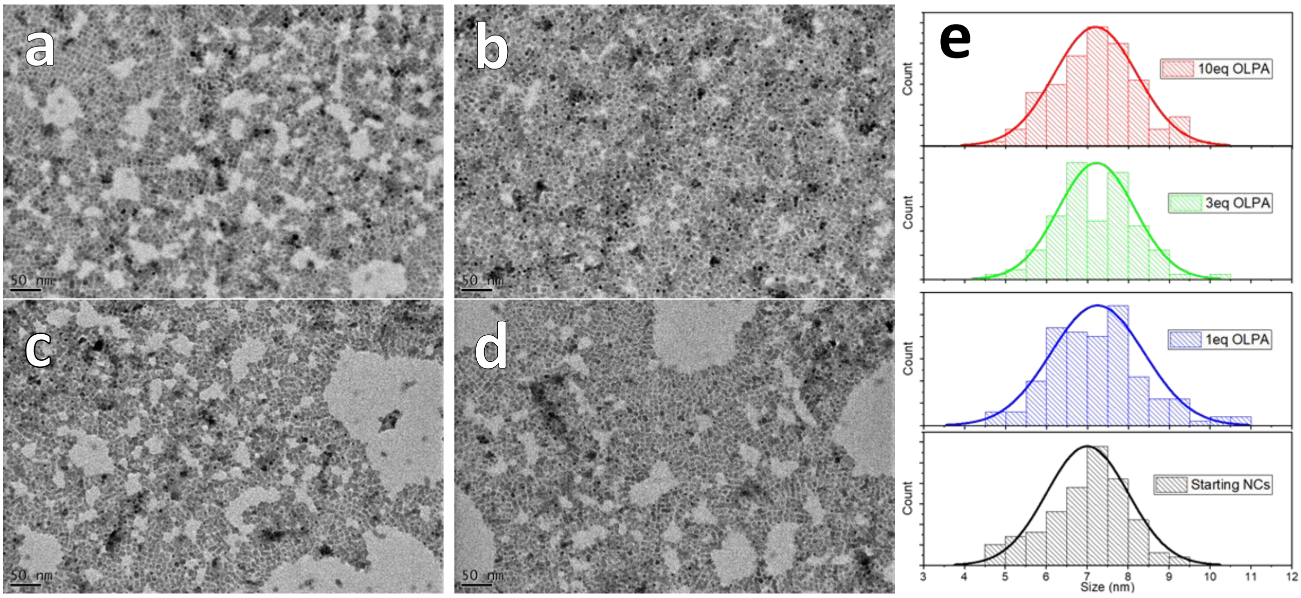

Figure S7. TEM images of (a) DDABr-capped NCs and those treated with either (b) 1 eq, (c) 3eq or (d) 10eq of OLPA after washing. The corresponding size histograms are reported in panel (e).

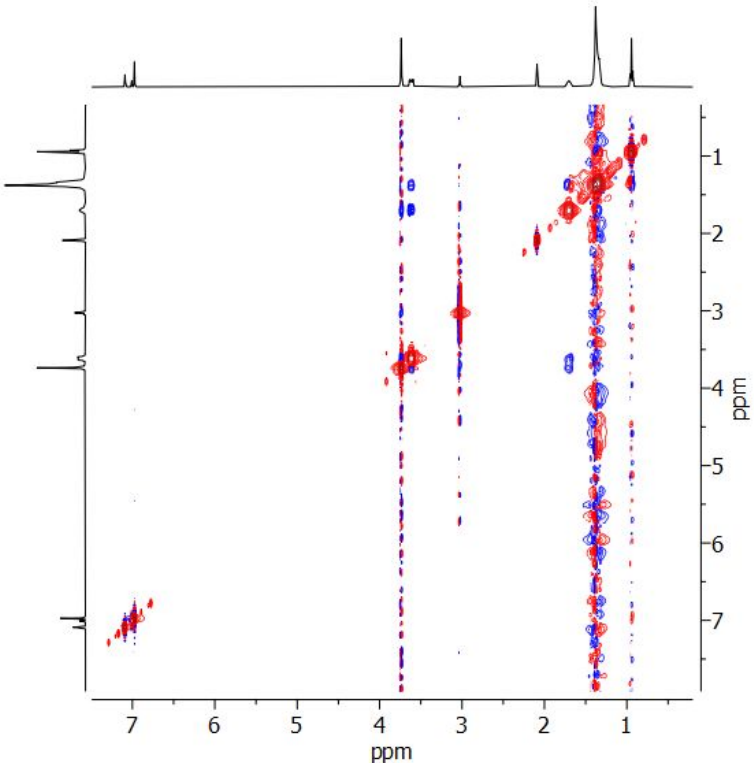

Figure S8. 2D ^{1}H-^{1}H NOESY experiment performed at 40°C of free DDABr molecules in toluene-d8. The free DDABr returns positive NOE (blue) cross peaks, characteristic of species with a short correlation times (τ_c). This temperature was employed in order to "break" the micelles that DDABr molecules otherwise form at room temperature (see J. Phys. Chem. B 2004, 108, 1, 438–443). At 300K, in fact, micelles return negative NOE (red) cross peaks in the 2D NOESY (characteristic of

species with a long τ_c), as reported in our recent work (see Figures S16-17 of *ACS Energy Lett.* 2019, 4, 4, 819–824).

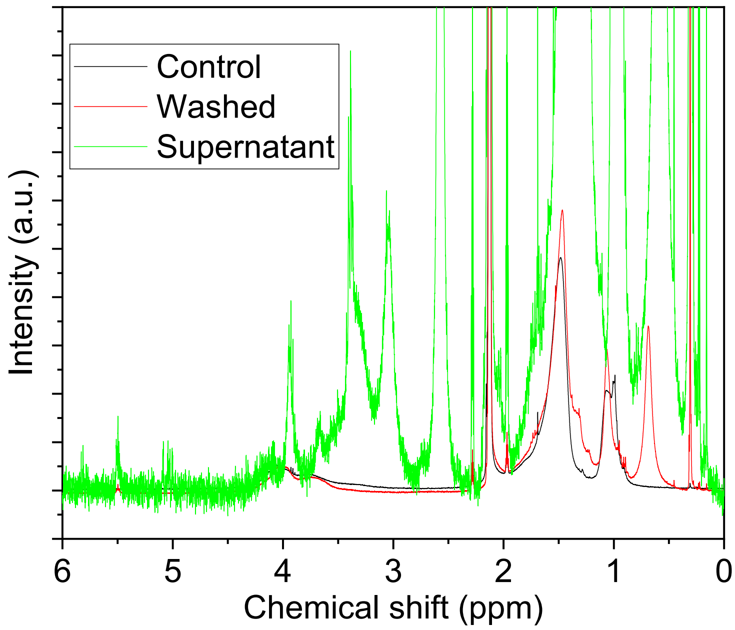

Figure S9. ^{1}H-NMR analysis of NC treated with 3eq of OLPA before (black curve) and after (red curve) a washing step with ethyl acetate (which consists in the addition of ethyl acetate, centrifugation and redispersion of the NCs in toluene-d8). The green curve is the ^{1}H-NMR analysis of the supernatant obtained in the washing step.

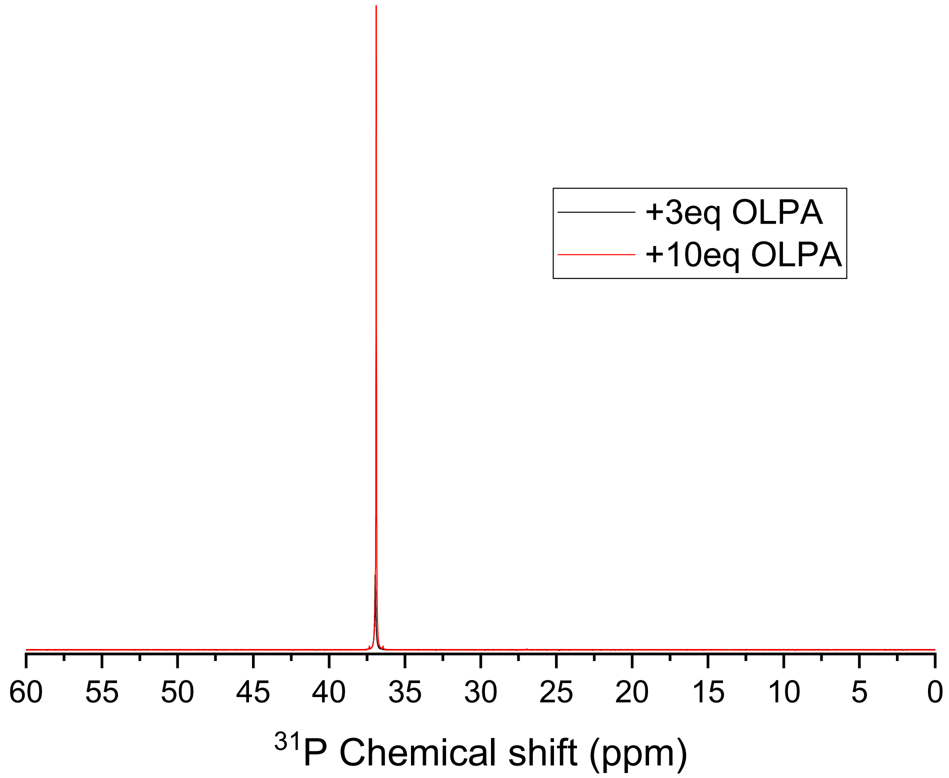

Figure S10. ^{31}P-NMR of 3eq OLPA (black line) and 10eq OLPA (red line) treated NCs after washing.

	Starting NCs	10eq OLPA treated
Cs (at%)	20.2	21.0
Pb (at%)	19.4	19.8
Br (at%)	53.8	53.8
N (at%)	6.5	5.4

Table S2. XPS result of starting DDAB caped NCs and NCs after 10 eq OLPA treatment.

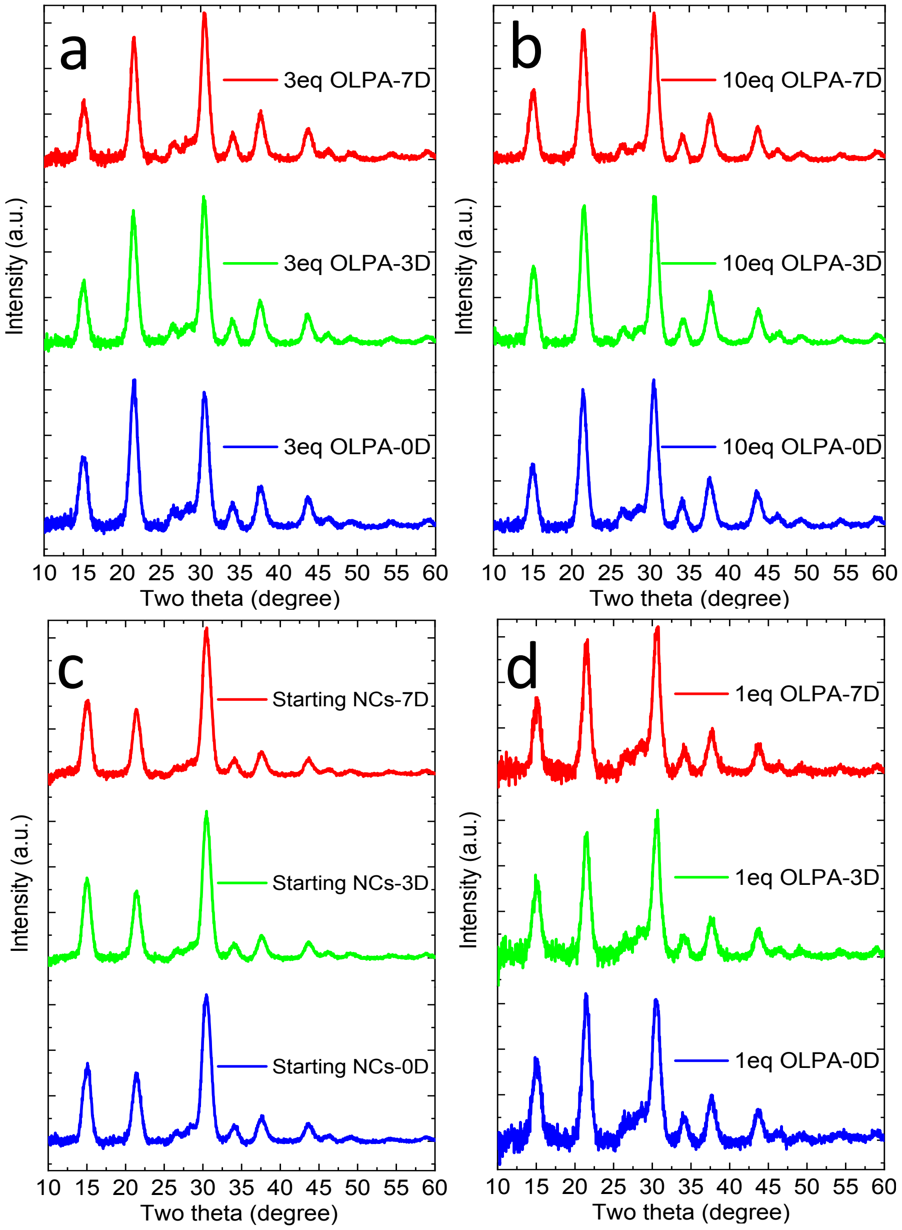

Figure S11. XRD patterns of (a) starting DDABr-capped NCs and those treated with either (b) 1eq, (c) 3eq or (d) 10eq of OLPA after air exposure for 0, 3 and 7 days (0D, 3D and 7D, respectively).

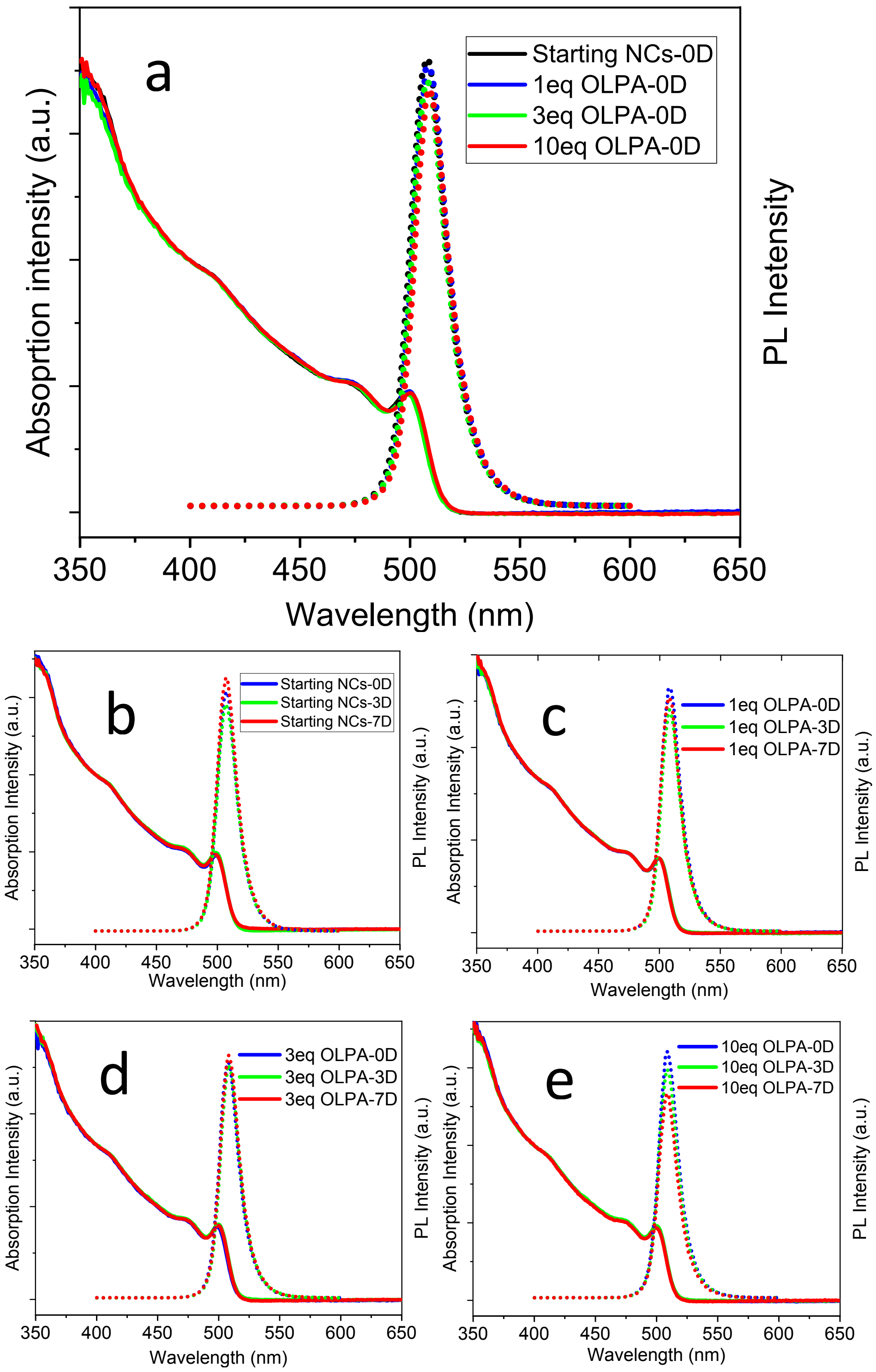

Figure S12. Absorption and PL spectra of (a) starting DDABr-capped NCs and those treated with either (b) 1eq, (c) 3eq or (d) 10eq of OLPA after air exposure for 0, 3 and 7 days (0D, 3D and 7D, respectively).

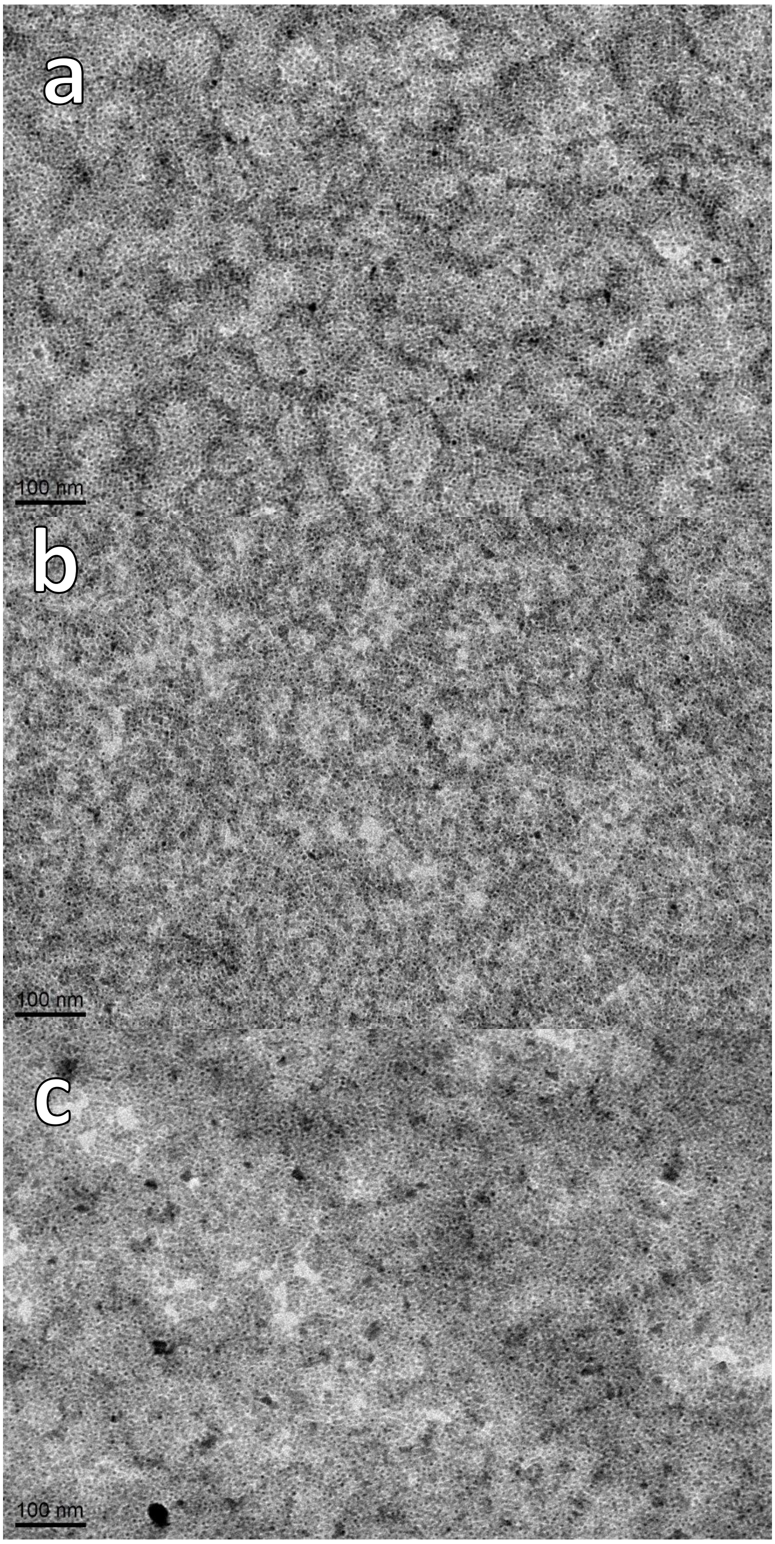

Figure S13. TEM images of (a) 1 eq HOA, (b) 3 eq HOA and (c) 10 eq HOA treated NCs after washing.

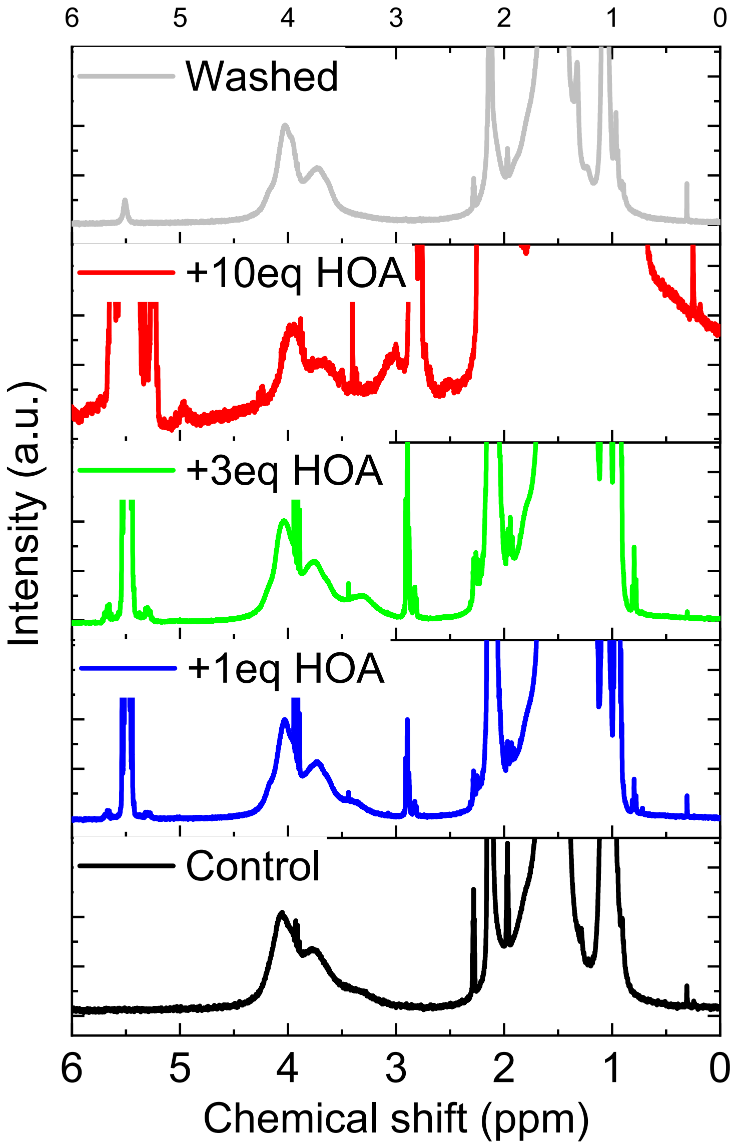

Figure S14. ^{1}H NMR analysis of DDABr-capped NCs, and 1 eq HOA, 3eq HOA, 10eq HOA treated NCs. The top panel shows the ^{1}H NMR analysis of NCs treated with 3eq of HOA and washed with ethyl acetate.

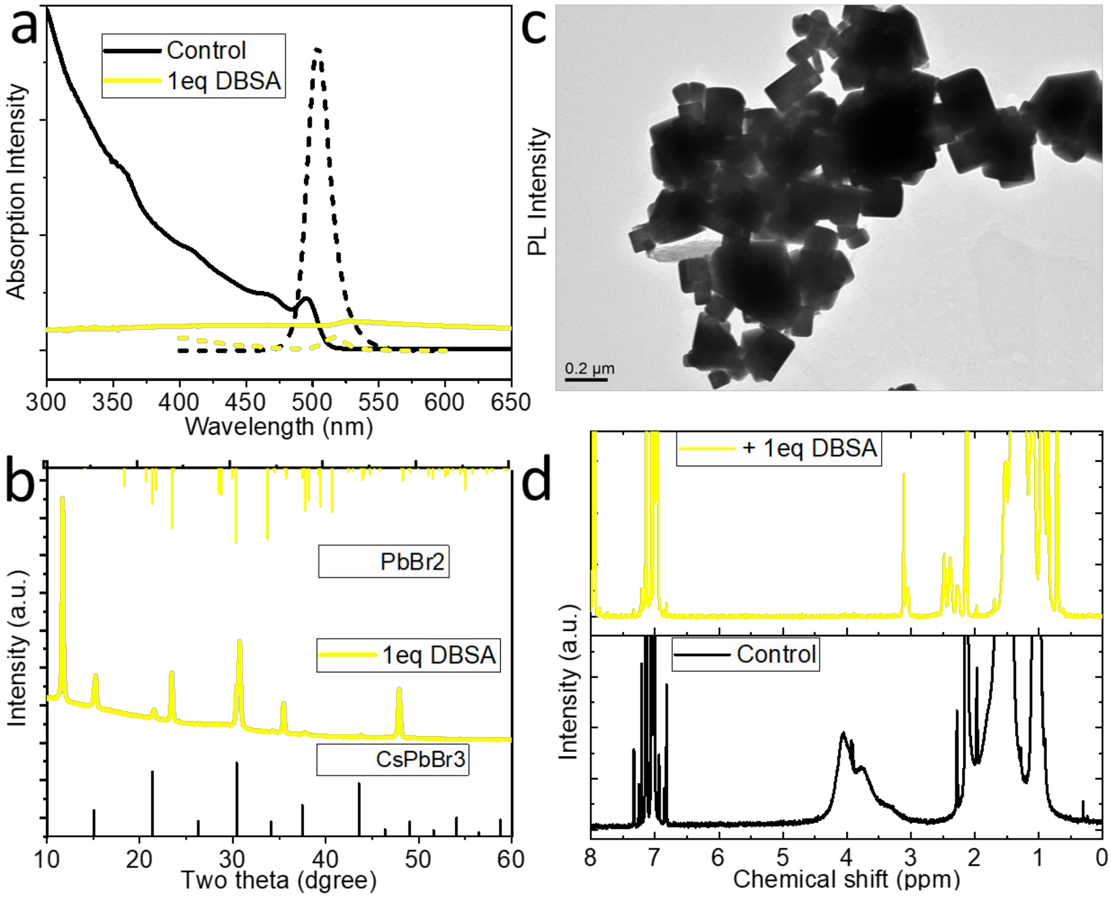

Figure S15. (a) Absorption and emission spectra, (b) XRD (c) TEM and (d) ^{1}H-NMR spectrum of DBSA treated NCs.

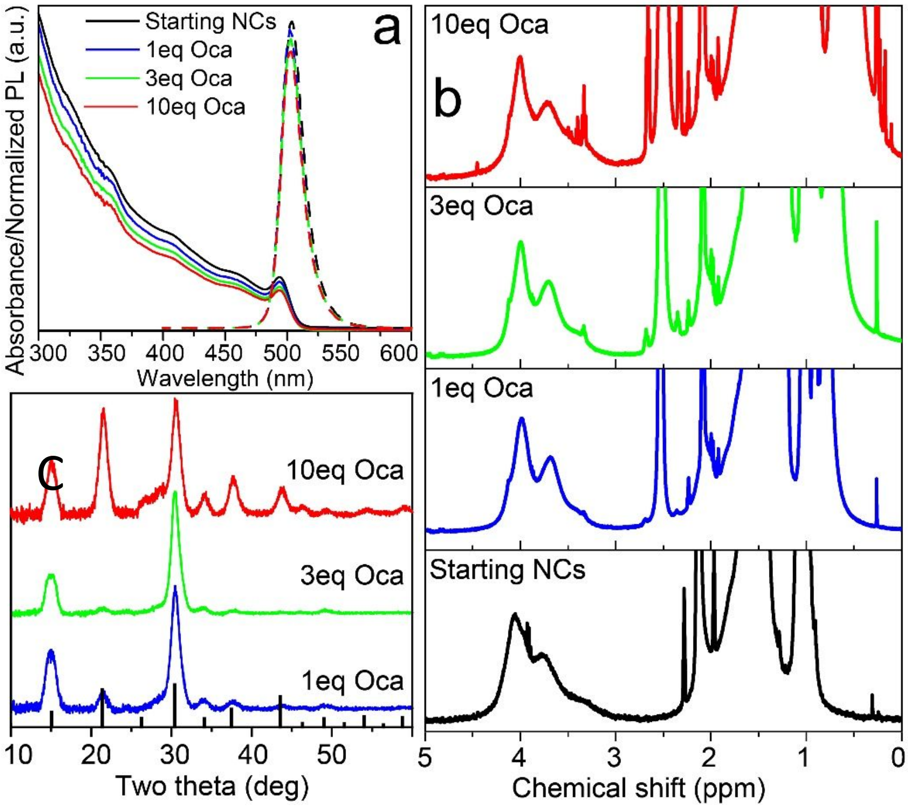

Figure S16. DDABr-capped $CsPbBr_3$ nanocrystals treated with 1-10 eq amount of degassed octylamine (C8B) and the corresponding (a) Absorption and PL intensity curves; (b) 1H-NMR; (c) XRD patterns.
